# Laminar dynamics of deep projection neurons and mode of subplate formation are hallmarks of histogenetic subdivisions of the human cingulate cortex before onset of arealization

**DOI:** 10.1007/s00429-022-02606-7

**Published:** 2023-01-02

**Authors:** Alisa Junaković, Janja Kopić, Alvaro Duque, Pasko Rakic, Željka Krsnik, Ivica Kostović

**Affiliations:** 1 School of Medicine, Croatian Institute for Brain Research, University of Zagreb, Zagreb, Croatia; 2 School of Medicine, Yale University, New Haven, CT 06510 USA

**Keywords:** Human brain development, Cerebral cortex, Subplate, Cingulate cortex

## Abstract

The cingulate gyrus, as a prominent part of the human limbic lobe, is involved in the integration and regulation of complex emotional, executive, motivational, and cognitive functions, attributed to several functional regions along the anteroposterior axis. In contrast to increasing knowledge of cingulate function in the adult brain, our knowledge of cingulate development is based primarily on classical neuroembryological studies. We aimed to reveal the laminar and cellular development of the various cingulate regions during the critical period from 7.5 to 15 postconceptional weeks (PCW) before the formation of Brodmann type arealization, employing diverse molecular markers on serial histological sections of postmortem human fetal brains. The study was performed by analysis of: (1) deep projection neuron (DPN) markers laminar dynamics, (2) all transient laminar compartments, and (3) characteristic subplate (SP) formation-expansion phase. We found that DPN markers labeling an incipient cortical plate (CP) were the first sign of regional differentiation of the dorsal isocortical and ventral mesocortical belt. Remarkably, increased width of the fibrillar marginal zone (MZ) towards the limbus, in parallel with the narrowing of CP containing DPN, as well as the diminishment of subventricular zone (SVZ) were reliable landmarks of early mesocortical differentiation. Finally, the SP formation pattern was shown to be a crucial event in the isocortical cingulate portion, given that the mesocortical belt is characterized by an incomplete CP delamination and absence of SP expansion. In conclusion, laminar DPN markers dynamics, together with the SVZ size and mode of SP formation indicate regional belt-like cingulate cortex differentiation before the corpus callosum expansion and several months before Brodmann type arealization.

## Introduction

The great limbic lobe (*le grand lobe limbique*) that encircles the brainstem and the great commissure corpus callosum (CC), introduced by French neurologist Paul Broca, and later explored by James Papez and Paul MacLean, was enigmatic since its discovery with little attention paid to the cytoarchitectonic differences in the cingulate gyrus (Broca 1878; Papez 1937; MacLean 1949, 1952; Pessoa and Hof 2015).

Functional neuroimaging made it obvious that the cingulate gyrus (cingulate cortex) is not a homogenous structure, but that it is comprised of several functional divisions. Vogt introduced the four-region neurobiological model that describes anatomically and functionally different regions of the cingulate cortex and according to this model, it is subdivided into the anterior cingulate cortex (ACC), corresponding to Brodmann areas (BA) 32, 24 and 25; midcingulate cortex (MCC); posterior cingulate cortex (PCC), corresponding to BA 31 and 23 and, retrosplenial cortex (RSC), located posteroinferiorly to the splenium of the CC and cytoarchitectonically referred to as BA 29 and 30 (Vogt 1993, 2009, 2019; Palomero-Gallagher et al. 2008; Vogt and Palomero-Gallagher 2012). We agree that Brodmann cytoarchitectonic maps (Brodmann 1909; Economo and Koskinas 1925) are not suitable for analyzing early regional cortical development, but for analyzing adult brains as stated by Filimonoff (1947). The cingulate gyrus is involved in the integration and regulation of complex emotional (mood states, maternal-infant interactions, social interactions) (Vogt 2005; Gholampour et al. 2020), executive (motor, vocalization, visceromotor) (Carter et al. 1999; Asemi et al. 2015), motivational (Touroutoglou et al. 2019), and cognitive (nociceptive, self-awareness and self-generated decisions) (Paus 2001; Vogt 2005; Maister et al. 2015; Lou et al. 2017; Falsaperla et al. 2022) functions attributed to several functional regions and hubs along the anteroposterior axis (ACC, MCC, and PCC) (Vogt and Palomero-Gallagher 2012; Catani et al. 2013; Rolls 2019). The anterior part of the cingulate gyrus is primarily involved in emotions and autonomic responses, MCC has primarily premotor functions due to its projections to the spinal cord, motor and premotor cortices, while the PCC and RSC are involved in memory and visuospatial orientation (Devinsky et al. 1995; Maddock et al. 2001; Vogt 2009, 2019).

The underlying structural foundation of these distinct functions was explained by differences in the architecture and connectivity of these areas (Palomero-Gallagher et al. 2008; Vogt and Palomero-Gallagher 2012). A review of allocortical portions was presented in great detail in the handbook of Stephan (1975) showing that the major part of the dorsal cingulate gyrus is isocortical (neocortical), first observed by Cajal (1911). Connectivity of the cingulate cortex with diverse cortical and subcortical structures was initially exquisitely shown by Yakovlev et al. (1960a,b,c,d) and Nauta (1972) who proposed the new limbic system concept where cingulate cortex as a part of the frontal lobe, is included in the limbic connectivity centered around hypothalamus (Nauta 1972).

After the introduction of functional neuroimaging, the operational significance of the cingulum fiber bundle, a massive compact white matter tract that runs off the cingulate cortex, was attributed to its contributions to the limbic system, as a variable and weakly defined system of clear clinical interest. The associative cingulum bundle is shown with diffusion tensor imaging (DTI) to be a major hub in the human brain connectome (Vasung et al. 2010; Bubb et al. 2018).

In this paper, the focus is on the role of deep projection neurons-DPN (cortical layer V and VI neurons), born before superficial layer neurons, and the phenomenon of the secondary expansion of the SP (second CP phase, SP formation phase) (Poliakov 1949; Kostovic and Rakic 1990; Duque et al. 2016). Based on our previous studies, we included all layers (compartments) of the future cerebral cortex in the analysis (*“compartmental approach”*), with a particular emphasis on all transient zones forming a cortical anlage: cortical zones (MZ, CP, SP) and proliferative zones (VZ and SVZ) (Meyer 2001; Bystron et al. 2008; Luhmann et al. 2018; Žunić Išasegi et al. 2018). Therefore, in this study, we selected a period between 7.5 and 15 PCW in the early fetal life when differentiation of the ventral and dorsal cingulate cortex occurs. Currently, there is a gap in knowledge on molecular mechanisms, as well as the cytoarchitectonic descriptions between the early cortex-type territorial parcellation and later-occurring areal differentiation. Importantly, numerous Hominini-specific genes are expressed during that time of development (Nowakowski et al. 2017). Moreover, the role of diverse genes is well examined in the late embryonic period, indicating a huge comparative and evolutionary significance. The late embryonic period requires more attention from the point of revealing expression of transcription factors, extracellular matrix (ECM) molecules, and additional DPN markers, given that supragranular layer neurons are not born yet. Previously, it was shown that limbic supracallosal cortex development occurs in a shorter period (Macchi 1951), and thus is more mature at birth than the lateral telencephalon.

Additionally, this study is also focused on the dorsoventral differentiation of the cingulate cortex before, during, and immediately after CC development. More specifically, we aimed to provide developmental data on the differences in the enigmatic laminar development of the ventral and dorsal portion of the cingulate gyrus in the ACC and PCC during early prenatal development. Previously described cytoarchitectonic differences were further correlated using DPN markers, in addition to glial, synaptic, fibrillar, and ECM markers. Therefore, we analyzed early regional differences in the medial interhemispheric cortex following the dynamics of neuronal laminar markers, i.e., markers of future projection neurons, and thus analyzed CP delamination and SP formation-expansion (Hevner et al. 2001; Bedogni et al. 2010; Duque et al. 2016). Given that dorsal and ventral cingulate gyrus are characterized by anteroposterior and dorsoventral structural and functional differences, one of our aims was to discover how and when these differences appear.

The prenatal development of the nonhomogeneous and complex cingulate gyrus system along the medial aspect of the hemisphere was studied during the early days of histology, as shown in the classical literature (His 1904; Hochstetter 1919; Macchi 1951; Kahle 1969; Stephan 1975). Early developmental differences between the anlage of three-layered archicortex, transitional mesocortex (Filimonoff 1947; Stephan 1975) and six-layered isocortex were described (Rose 1926; Filimonoff 1947; Kahle 1969; Stephan 1975), but the debate remains as to what is the major substrate of these differences. Certainly, it was shown that the MZ and CP width serves as a key landmark in distinguishing the cingulate gyrus before the appearance of the cingulate sulcus, CC, and supragranular cortical layers (Rakic and Yakovlev 1968; Kostović and Krmpotić 1976). Additional criteria were later proposed by Kostović et al. (2004), who found differential distribution in synaptic arrangement and neural morphology from the dorsal to the ventral cingulate cortex. Also, Kostović and Krmpotić (1976) proposed that the “second cortical plate” (Poliakov 1949), subplate formation (SPf), and differences in the SP secondary expansion, may explain differences between the ventral mesocortical and the dorsal isocortical parts of the cingulate gyrus (Kostović and Krmpotić 1976; Kostović et al. 2004).

The cingulate cortex develops very early, like other parts of the limbic cortex (Rakic and Nowakowski 1981) and it participates in active resting state networks in utero in vivo (Doria et al 2010) which from early on are involved in behavioral and emotional neural networks. Hence, besides understanding the functional anatomy of the cingulate cortex in the adult human brain, it is crucial to understand the prenatal development of the human cingulate cortex, if we want to understand how it may participate in the later pathogenesis of diverse neurodevelopmental disorders, such as autism spectrum disorder-ASD (Mundy 2003; Simms et al. 2009), obsessive–compulsive disorder and behavior (McGovern and Sheth 2017), sociopathic behavior, diminished self-awareness, bipolar disorder (Kowatch et al. 2003), and schizophrenia (Yücel et al. 2002; Leech and Sharp 2014) among others. The cingulate cortex is an excellent model to study the laminar differential development of basic cortical types (iso-, archi-, meso-cortex).

We hypothesize that DPN markers show cortex-type specific regional differentiation during the early fetal period and the final pattern of differences between main cortical types is established during the SP formation. We expect that simultaneous analysis of proliferative markers in VZ/SVZ and projection neuron markers in CP and SP will give us an answer, at least topographically grounded, on how dorsoventral and anteroposterior differences in the cingulate cortex develop.

These new findings will provide an insight into the early development of the medial interhemispheric cingulate cortex through the early and mid-fetal period (from 7.5 to 15 PCW), with a special emphasis on the SP formation and expansion (13–15 PCW), as a key human specific neurodevelopmental event. Given that the SP formation-expansion period is typical for the six-layered isocortex, we think that only a study focused on the SP formation period can provide an answer to differences in dorsal vs. ventral cingulate cortex development.

## Materials and methods

### Human fetal brain tissue

Human brain material is part of the Zagreb Neuroembryological Collection (Kostovic et al. 1991), obtained during regular autopsies after spontaneous or medically indicated abortions at clinical hospitals affiliated with the University of Zagreb, School of Medicine. All specimens were without macroscopic or microscopic central nervous system pathology. A sampling of the tissue was performed following the Declaration of Helsinki (2000) and approved by the Internal Review Board of the Ethical Committee of the School of Medicine, University of Zagreb. After extraction during the autopsy, postmortem human brains were immersion-fixed in 4% paraformaldehyde (PFA) in 0.1 M phosphate-buffered saline (PBS; pH = 7.4). The fetal age was determined based on the pregnancy records and crown-rump length (CRL) in millimeters and expressed in PCWs, instead of gestational weeks (GW). Following fixation, tissue blocks were embedded in paraffin and sectioned in a coronal or semi-horizontal plane on a microtome (Leica, SM2000R, Wetzlar, Germany) at 10 to 20 µm thick sections. In total, seven brain specimens were systematically processed and analyzed in the period from 7.5 to 15 PCW (CRL 28–120 mm). From these seven, three human fetal brain specimens aged 8, 13 and 15 PCW were serially sectioned from the frontal to the occipital pole to follow the anatomy of the whole cingulate gyrus and then processed by immunohistochemistry. The prospective cingulate cortex is located between the folded archicortical dorsal hippocampus ventrally and the isocortex (neocortex) dorsally (Kostović and Krmpotić 1976) and we analyzed the medial interhemispheric cortex.

### Immunohistochemistry (IHC) and immunofluorescence (IF) on human postmortem prenatal brain tissue

Classical histological Cresyl violet (Nissl) staining in the 0,5% Cresyl violet solution at room temperature was used to demonstrate different cytoarchitectonic cortical compartments and to allow comparisons of adjacent sections. PAS-Alcian Blue histochemistry was used to demonstrate the abundant ECM predominantly in the SP compartment. Histological stainings were performed according to the previously described protocols (Kostovic and Goldman‐Rakic 1983).

Immunohistochemistry was done according to our standard protocol described by Žunić Išasegi et al. (2018). The first step is deparaffinization of the paraffin-embedded tissue sections in Xylol solution. Following, slides were immersed in 100% EtOH (2 × 5 min), 96% EtOH (2 × 5 min), and 70%EtOH (1 × 5 min) and rinsed in the 1xPBS for 10 min. Antigen retrieval was done by boiling sections in a citrate buffer (pH = 6.0). Following the antigen retrieval process, sections were pretreated with methanol (MetOH) and H_2_O_2_ for 30 min to block the endogenous peroxidase activity. Afterward, sections were rinsed and incubated in the blocking solution (5% bovine serum albumin-BSA and 0, 5% Triton X-100 in 1xPBS) for 1 h. Primary antibodies were incubated at 4 °C during the night. We used the following primary antibodies: glial markers anti-GFAP and anti-vimentin, fibrillar anti-SMI312, synaptic and axonal (anti-SNAP25, anti-SYN), projection neuron markers (anti-Tbr1, anti-CTIP2, anti-TLE4, anti-SOX5), ECM marker (anti-NCAN), proliferative (anti-Ki67), stem cell marker (anti-SOX2), a marker of Cajal-Retzius cells (anti-Reelin), intermediate progenitor cell marker (anti-Tbr2) and microtubular marker (anti-TUBB3). For details about primary antibodies, please refer to Table 1. The next day, following rinsing in the 1xPBS, corresponding secondary biotinylated antibodies (Vectastain ABC kit, Vector Laboratories) were used. After rinsing in 1xPBS, sections were incubated with the Avidin–biotin peroxidase complex for 1 h, and staining was visualized with 3,3-diaminobenzidine (DAB) with metal enhancer (Sigma) or without metal enhancer (ImmPACT DAB EqV). Finally, sections were mounted and coverslipped. Immunofluorescence was done according to a standard protocol, similar to IHC, except that secondary antibodies were conjugated with fluorophores (AlexaFluor 555 and AlexaFluor 488), and the TrueBlack quencher (Biotium) was used. Slides were mounted with a mounting medium with nuclear stain DAPI (Vectashield with DAPI) and coverslipped. Images were visualized and analyzed using a high-resolution digital slide scanner NanoZoomer 2.0 RS (Hammamatsu, Japan).

**Table 1:** Antibodies used for the immunohistochemistry and immunofluorescence on the postmortem formalin-fixed paraffin embedded (FFPE) human brain specimens

Antibody	Supplier and catalogue number	RRID	Host	Antibody concentration
Anti-Vim	Dako, M7020	AB_2304493	Mouse monoclonal	1:100
Anti-GFAP	Dako, Z0334	AB_10013382	Rabbit polyclonal	1:500
Anti-SMI312	BioLegend, 837904	AB_2566782	Mouse	1:1000
Anti-SNAP25	BioLegend, 836304	AB_2566521	Mouse monoclonal	1:1000
Anti-SYN	Dako, M7315	AB_2687942	Mouse monoclonal	1:40
Anti-Tbr1	Abcam, ab31940	AB_2200219	Rabbit polyclonal	1:400
Anti-CTIP2	Abcam, ab18465	AB_2064130	Rat monoclonal	1:500
Anti-TLE4	Santa Cruz biotechnology, sc-365406	AB_10841582	Mouse monoclonal	1:50
Anti-SOX5	Abcam, ab94396	AB_10859923	Rabbit polyclonal	1 μg/mL
Anti-NCAN	Sigma, HPA036814	AB_10673666	Rabbit polyclonal	1:500
Anti-Ki67	Dako, M7240	AB_2142367	Mouse monoclonal	1:50
Anti-SOX2	SigmaAldrich, AB5603	AB_2286686	Rabbit polyclonal	1:200
Anti-Reelin	Millipore, MAB5366	AB_2285132	Mouse monoclonal	1:1000
Anti-Tbr2	Abcam, ab23345	AB_778267	Rabbit	1:200
Anti-TUBB3	Sigma, AMAb91394	AB_2716670	Mouse	1:500

## Results

Results are presented in a systematic manner describing all cortical compartments, namely, proliferative zones: VZ and SVZ, and cortical zones: MZ, CP, and SP.

Our focus was on two developmental phases—an early phase before the expansion of the subplate (7.5–10 PCW), and a phase during SP formation—expansion (13–15 PCW). More specifically, we described dorsoventral and anteroposterior differences during the formation of the cingulate cortex using the compartmental approach (analysis of all transient cortical compartments) by Nissl, T-box brain 1 (Tbr1), as well as analyzing additional molecular markers.

### Initial differentiation into cortex-type specific cingulate cortex belts-arcs (7.5–10 PCW)

The medial interhemispheric cortex is composed of the dorsal and ventral portion of the cingulate cortex and the hippocampal (archicortical) anlage. The dorsal portion of the prospective cingulate cortex has characteristics of isocortex-neocortex (thick CP and complete SP expansion), while the ventral portion of the cingulate cortex has mesocortical (allocortical) characteristics-thinner CP and SVZ, and incomplete SP expansion.

**Fig. 1:**
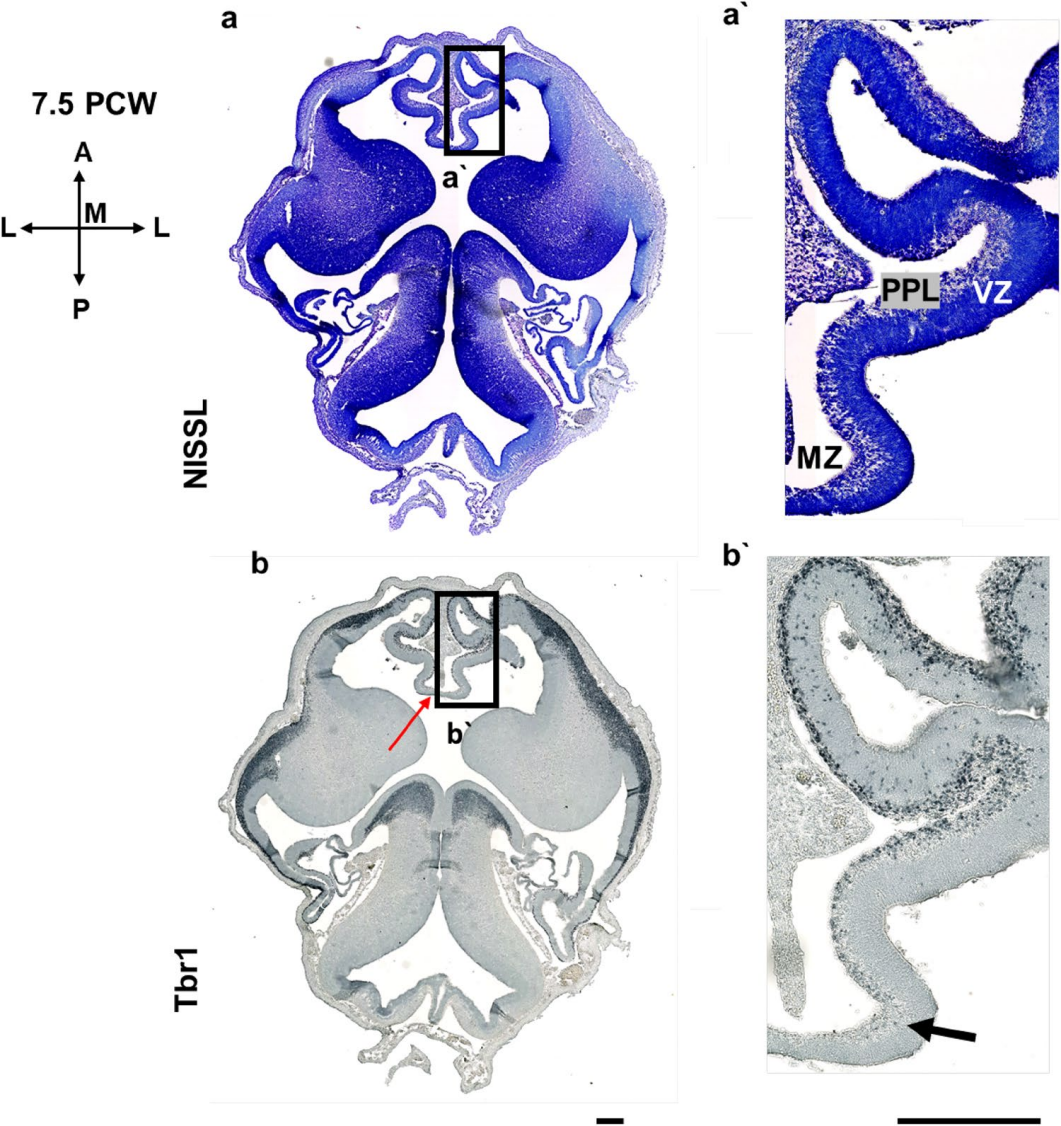
Development of the medial interhemispheric cortex revealed by Nissl staining (**a, a’**), and Tbr1 immunohistochemistry (**b, b’**) at 7.5 PCW (Carnegie stage 22), semihorizontal section. **a’** and **b’** represent the dorsal limbus (prospective cingulate cortex). Cortical plate is not yet developed. The section is tangentional, showing the first postmigratory Tbr1 positive neurons already within the PPL. In the archicortex (hippocampal limbus) no Tbr1 positive cells are observed (black arrow). The connection between the two limbus is marked with red arrow and it may correspond to the tissue bridge described as the precallosal “sling”. At this stage of development, there are no anteroposterior differences in the prospective cingulate cortex. *MZ* marginal zone, *VZ* ventricular zone, *PPL* preplate. Scalebar: 100 μm

Regional differences between the dorsal isocortex (neocortex) and medial limbic interhemispheric cortex can already be appreciated during early fetal brain development. Anteroposterior differences of the prospective cingulate gyrus are not yet developed at the earliest stage examined (7.5 PCW) (Fig. 1). At 7.5 PCW apparent transient zones, VZ and preplate (PPL) are observed, even though according to our results, proliferative zones are not yet easily separated. Instead, there is only one broad proliferative zone. Importantly, at this age, the CP is not developed either. It is obvious that the first postmigratory Tbr1 positive cells are already within the PPL, while the cells of the hippocampal (archicortical) anlage are not Tbr1 immunoreactive (Fig. 1).

**Fig. 2:**
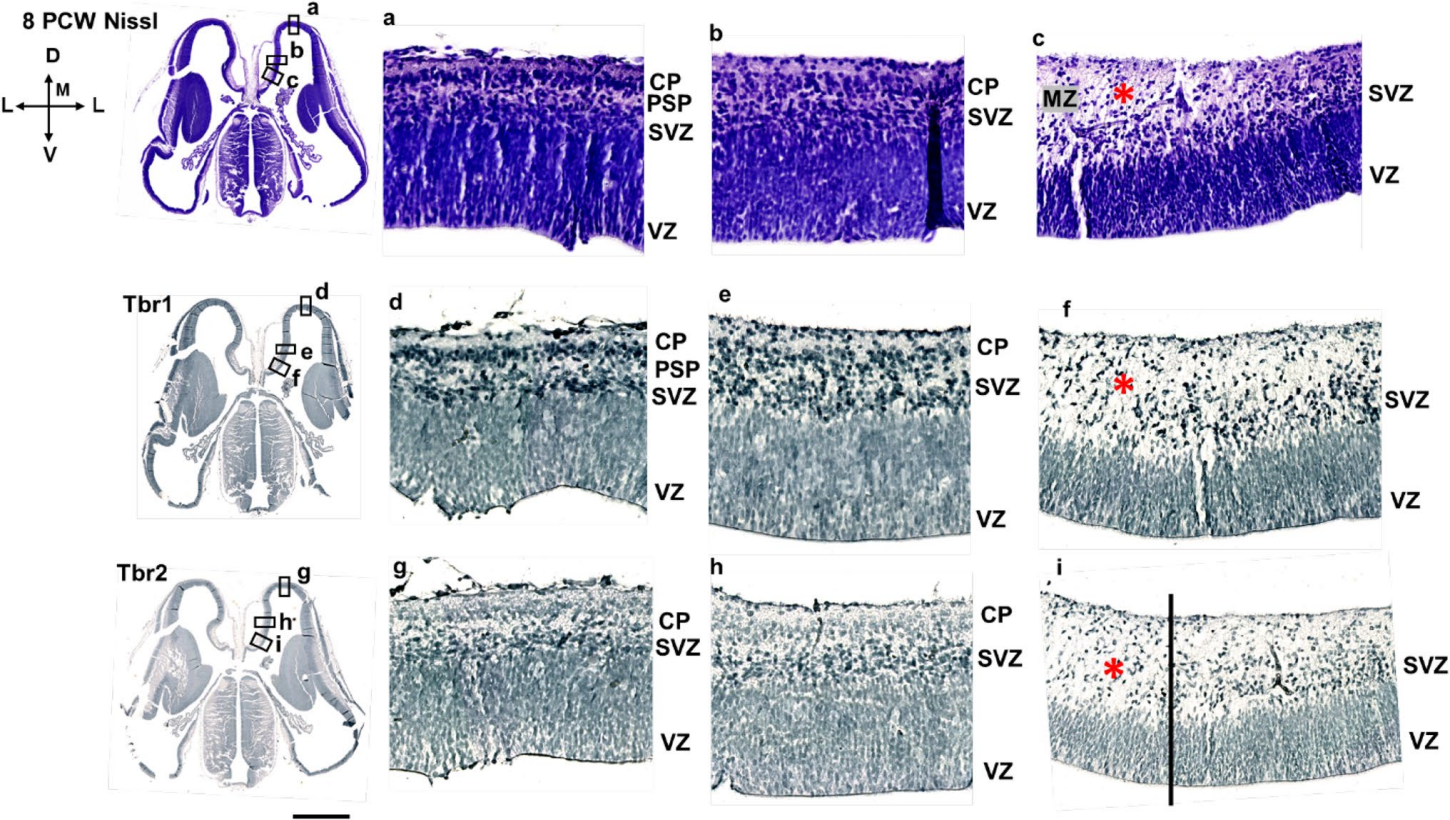
Development of the medial interhemispheric cortex revealed by Nissl staining, Tbr1 and Tbr2 immunohistochemistry at 8 PCW (Carnegie stage 23), coronal section, intermediate level. Tbr1 (**d, e, f**), projection neuron marker is expressed in the CP and SVZ. Tbr2 (**g, h, i**), an intermediate progenitor cells marker is predominantly expressed in the SVZ intermediate progenitors. The archicortex (**c**) has typically enlarged MZ (red asterisk) and underdeveloped CP. Thinner SVZ ventrally in the archicortical portion (hippocampal anlage) is serving as a crucial delineation landmark of neocortex, mesocortex and archicortex. The termination of the SVZ (diminished Tbr2 expression) in the archicortex (black line, i) marks the potential border between the meso- and archi-cortex. *MZ* marginal zone, *CP* cortical plate, *PSP* presubplate, *SVZ* subventricular zone, *VZ* ventricular zone, *A* dorsal neocortex, *B* mesocortex, *C* archicortex. Scalebar: 100 μm

**Fig. 3:**
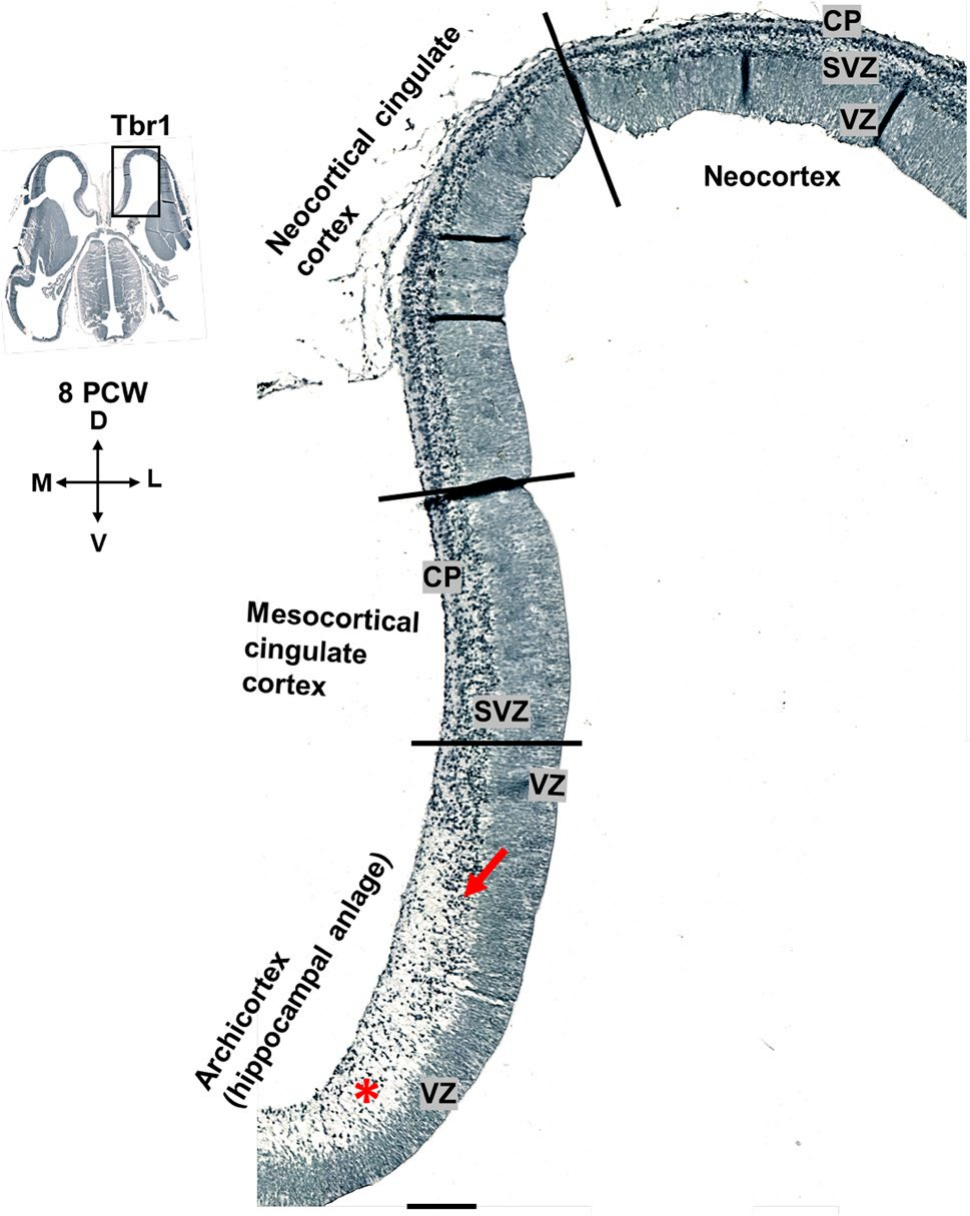
Dorsoventral gradient of the Tbr1 expression at 8 PCW (Carnegie stage 23), coronal section, intermediate level. Please note that this figure is the same section as Fig. 2 with greater details. Tbr1 is predominantly expressed in the CP and SVZ at 8 PCW. CP is already developed in neocortex, but not in the archicortex. In the archicortex, MZ is enlarged (red asterisk) and the VZ and SVZ are gradually thinner (red arrow). *MZ* marginal zone, *CP* cortical plate, *SVZ* subventricular zone, *VZ* ventricular zone. Scalebar: 500 μm

**Fig. 4:**
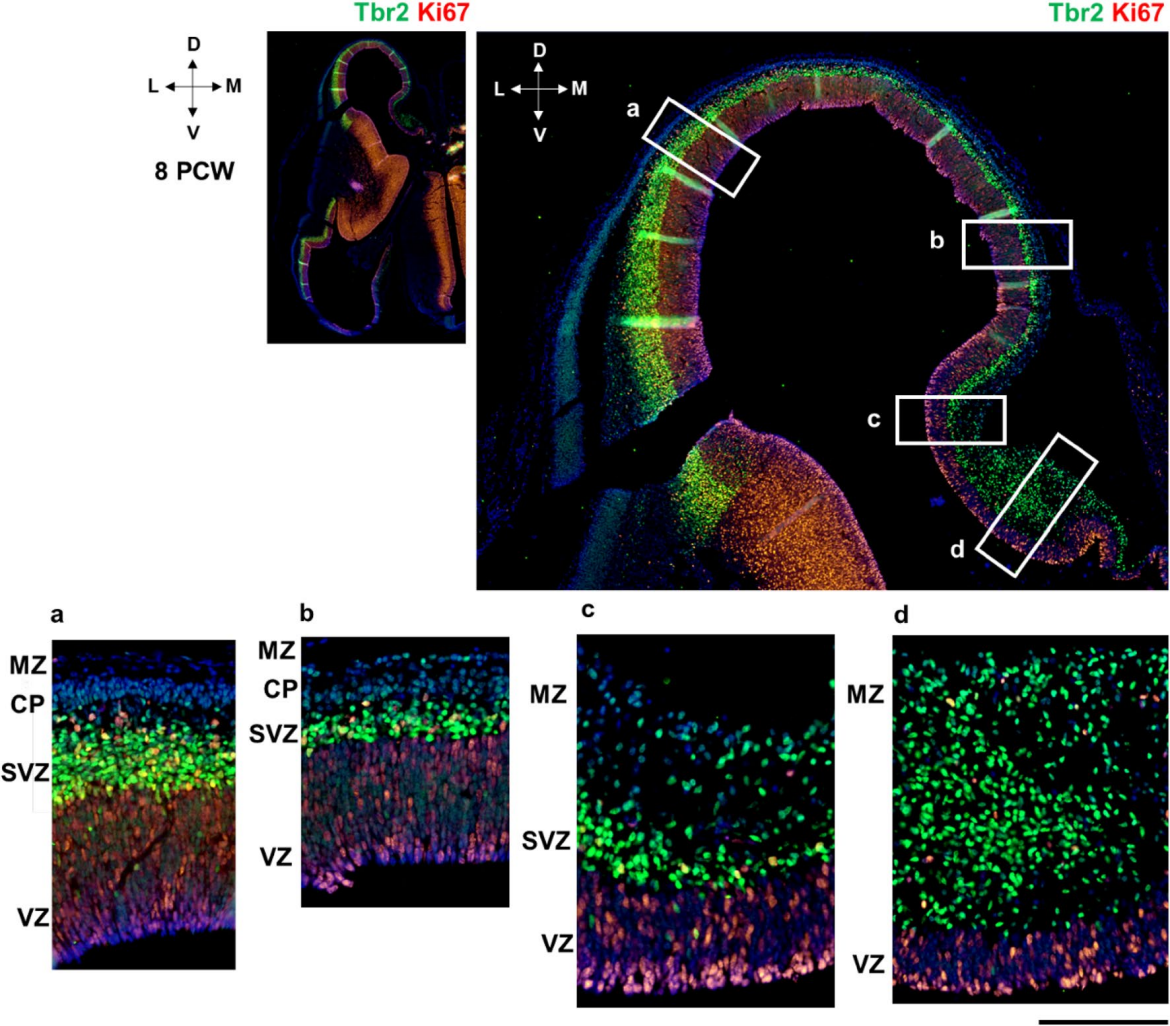
Thinning of the SVZ towards the archicortical limbus at 8 PCW (Carnegie stage 23), coronal section, intermediate level. Tbr2 positive SVZ (green) is narrowing towards the limbus. Ki67 positive cells (red) are observed in the proliferative zones and enlarged archicortical MZ (**d**). *MZ* marginal zone, *CP* cortical plate, *SVZ* subventricular zone, *VZ* ventricular zone. Scalebar: 100 μm

The first anteroposterior differences emerge during the formation of the initial CP at 8 PCW (Fig. 2). The future mesocortex shows some transitional features, e.g., less dense initial CP than in the isocortex. However, the presence of the pioneering CP already distinguishes mesocortical from the archicortical portion, characterized by an enormous MZ and the absence of the CP. Note that Tbr1 is mostly expressed in the SVZ and CP in the early fetal phase and evidently the CP is thinner in the mesocortical portion of the cingulate cortex than in the isocortical part (Fig. 2). In addition, the mesocortical part of the cingulate cortex has wider MZ than the isocortical part (Fig. 2a, b). At 8 PCW, Tbr1 is expressed in the SVZ and CP, and CP is gradually thinning towards the archicortex where it is absent (Fig. 3). The potential anatomical border between the mesocortical cingulate cortex and the archicortex could represent the wedge-shaped termination of the SVZ (inner SVZ-iSVZ) where Tbr2 shows diminished expression (Fig. 4). Note that SVZ is present during the early fetal phase (8 PCW) and Tbr2 positive SVZ is narrowing towards the limbus (Fig. 4), while CELF1 (Popovitchenko et al. 2020), delineates the CP and its gradual narrowing in the medial cortex towards its archicortical part (not shown).

**Fig. 5:**
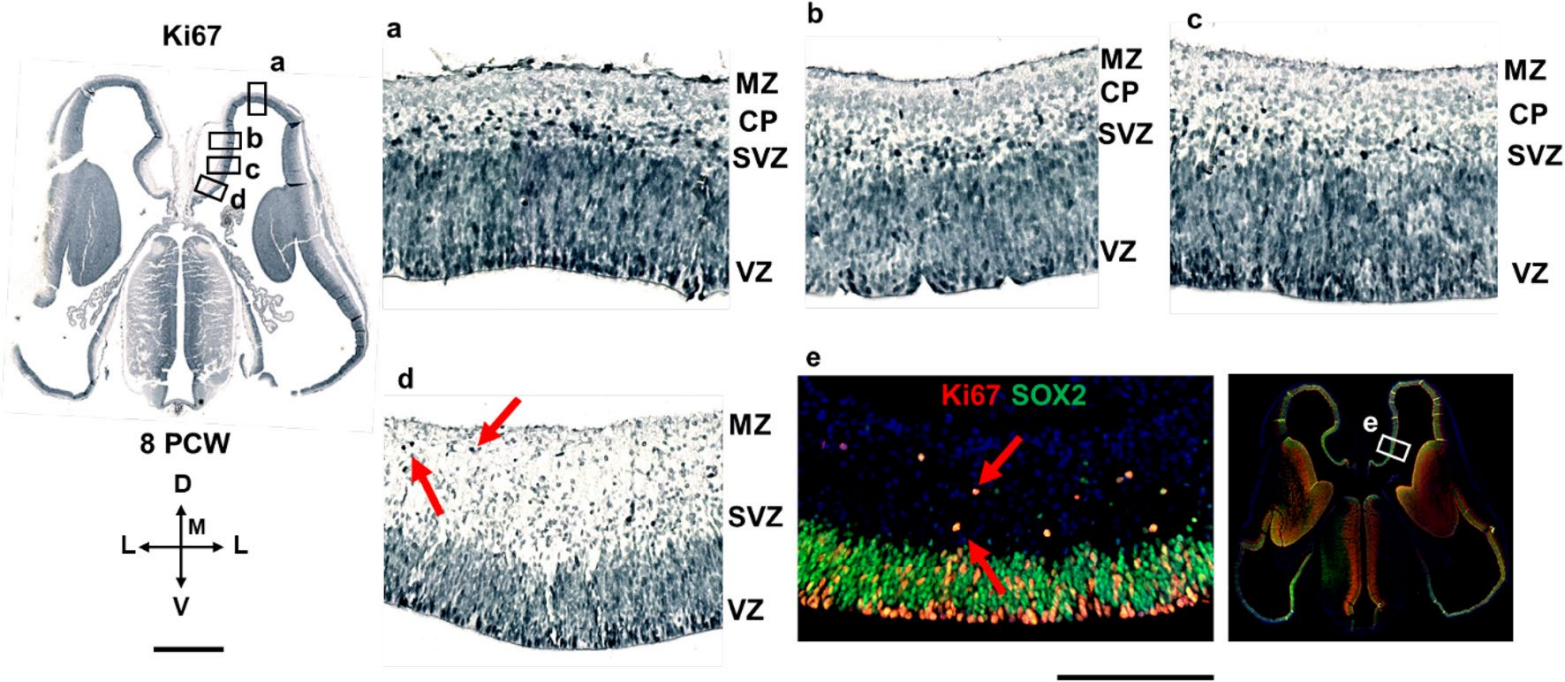
Proliferation in the medial interhemispheric cortex revealed by a proliferative marker Ki67 at 8 PCW, coronal section, intermediate level. Ki67 expression is extensive in the proliferative VZ and SVZ due to intense mitotic activity. In the hippocampal anlage (**d, e**), Ki67 positive cells (red arrows) are observed in the typically enlarged MZ, suggesting in situ proliferation in the archicortical MZ (**d**). Archicortical characteristics include the absence of the CP and SVZ, in addition to Ki67 positive proliferative cells in the MZ. SOX2 positive (green) stem cells are predominantly in the VZ (**e**). *MZ* marginal zone, *CP* cortical plate, *SVZ* subventricular zone, *VZ* ventricular zone. Scalebar: 500 μm, 100 μm

The ventral border of the prospective cingulate cortex can be roughly estimated due to significant enlargement of the MZ, underdeveloped CP, and less voluminous VZ and SVZ (iSVZ). SVZ (iSVZ) is shown by the intermediate progenitor cells marker Tbr2 (Fig. 4). Archicortex has no visible SVZ, but shows abundant Tbr2 positive cells (Fig. 4d). Proliferative marker Ki67 is expressed in the typically enlarged archicortical MZ suggesting in situ cellular proliferation in the MZ (Fig. 5). In addition, we observed SOX2 positive stem cells predominantly in the VZ, whereas a proliferative marker Ki67 is expressed in the proliferative zones and MZ (Figs. 4 and 5).

**Fig. 6:**
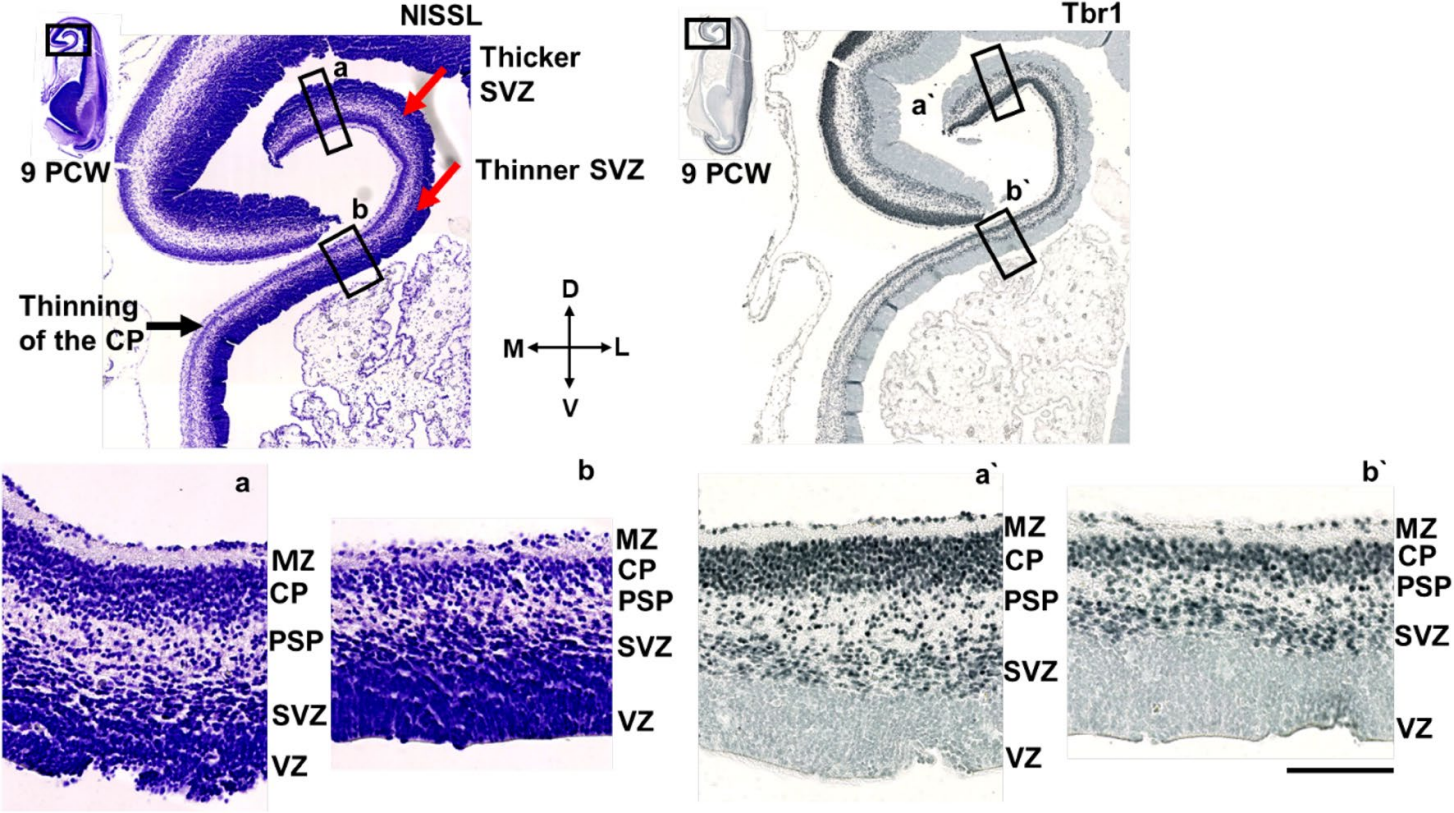
Medial interhemispheric cortical development revealed by Nissl staining and Tbr1 immunohistochemistry at 9 PCW, coronal sections. All transient cortical compartments (MZ, CP, PSP, SVZ, VZ) are easily distinguished. In the medial interhemispheric cortex, CP and SVZ are becoming gradually thinner towards the archicortex (**a, b**). Tbr1 (**a’, b’**) is expressed in the CP and SVZ. Please note a broken continuity between two parts of the medial cortex. *MZ* marginal zone, *CP* cortical plate, *PSP* presubplate, *SVZ* subventricular zone, *VZ* ventricular zone. Scalebar: 100 μm

In the medial interhemispheric cortex at 9 PCW (Fig. 6), CP and SVZ become thinner in the dorsoventral gradient towards the limbus. According to our findings, thinning of the CP, PSP, and SVZ and typical MZ enlargement could delineate the transitional mesocortical anlage of the cingulate cortex. At 10 PCW, Tbr1 positive CP is located between the two fibrillar/synaptic layers (MZ and PSP/IZ), narrowed in the medial limbic cortex, and absent in the archicortex (not shown).

### Regional ventrodorsal and anteroposterior differences between specific cortical patterns became established during the subplate expansion period (13–15 PCW)

**Fig. 7:**
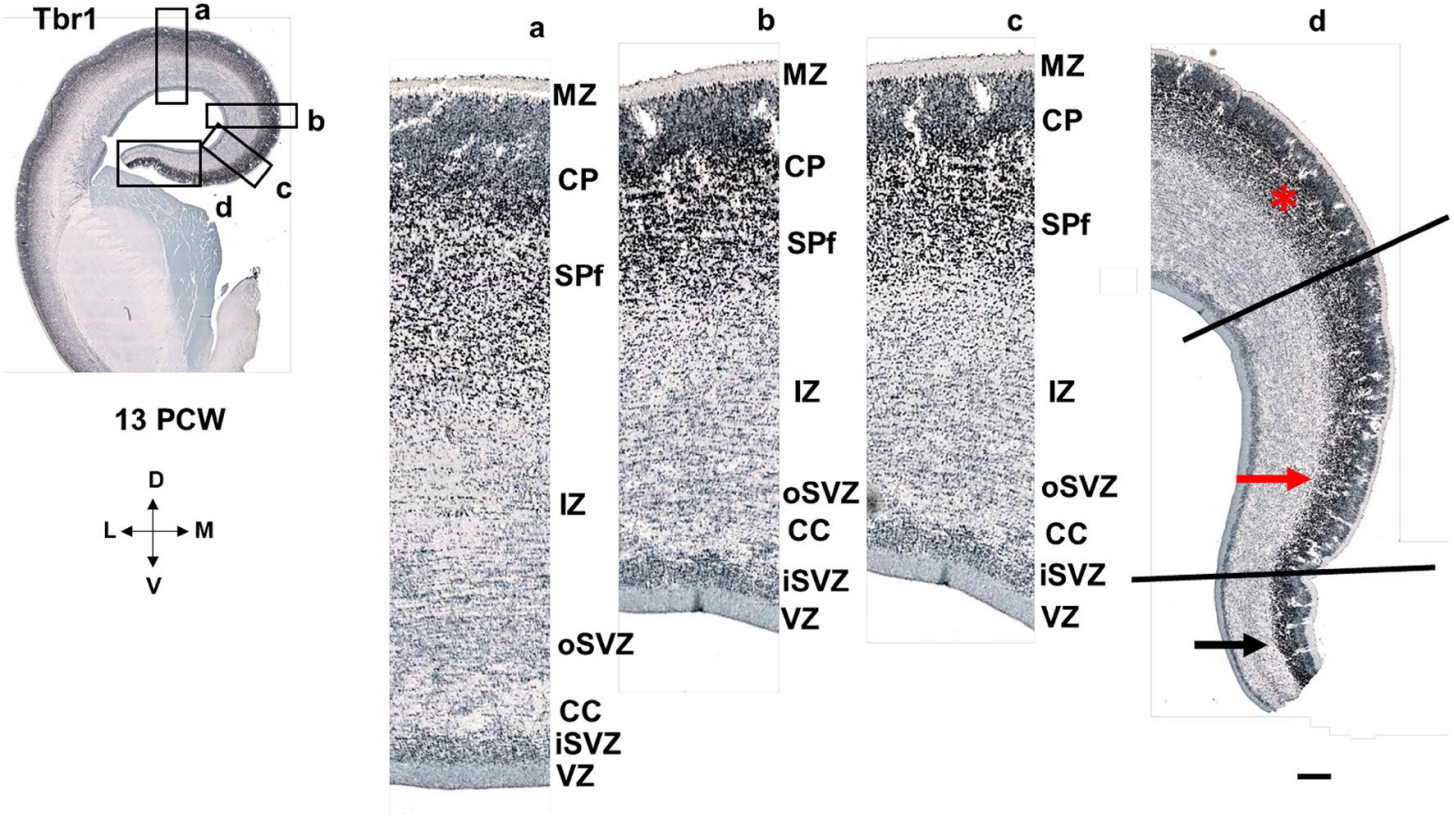
Subplate formation period revealed by the deep projection neuron marker Tbr1 at 13 PCW, coronal sections, anterior part. Rectangles a, b, c, and d represent the dorsal isocortex (**a**), medial isocortex (**b**) and parts of the cingulate cortex (**c, d**) with magnified images. SP is formed from the deep portion of the CP in the dorsal isocortex, as well as in the dorsal isocortical part of the cingulate cortex (marked with red asterisk). Incomplete SP expansion is observed in the mesocortical ventral cingulate cortex (red arrow), while the archicortical part is characterized by the absence of CP delamination-absence of SP expansion (black arrow). Black lines define the borders between complete, incomplete and absent SP expansion (CP delamination). Corpus callosum is formed, seen as callosal fibers between iSVZ and oSVZ. *MZ* marginal zone, *CP* cortical plate, *SPf* subplate in formation, *SVZ* subventricular zone, *iSVZ* inner SVZ, *oSVZ* outer SVZ, *VZ* ventricular zone, *CC* corpus callosum. Scalebar: 100 μm

**Fig. 8:**
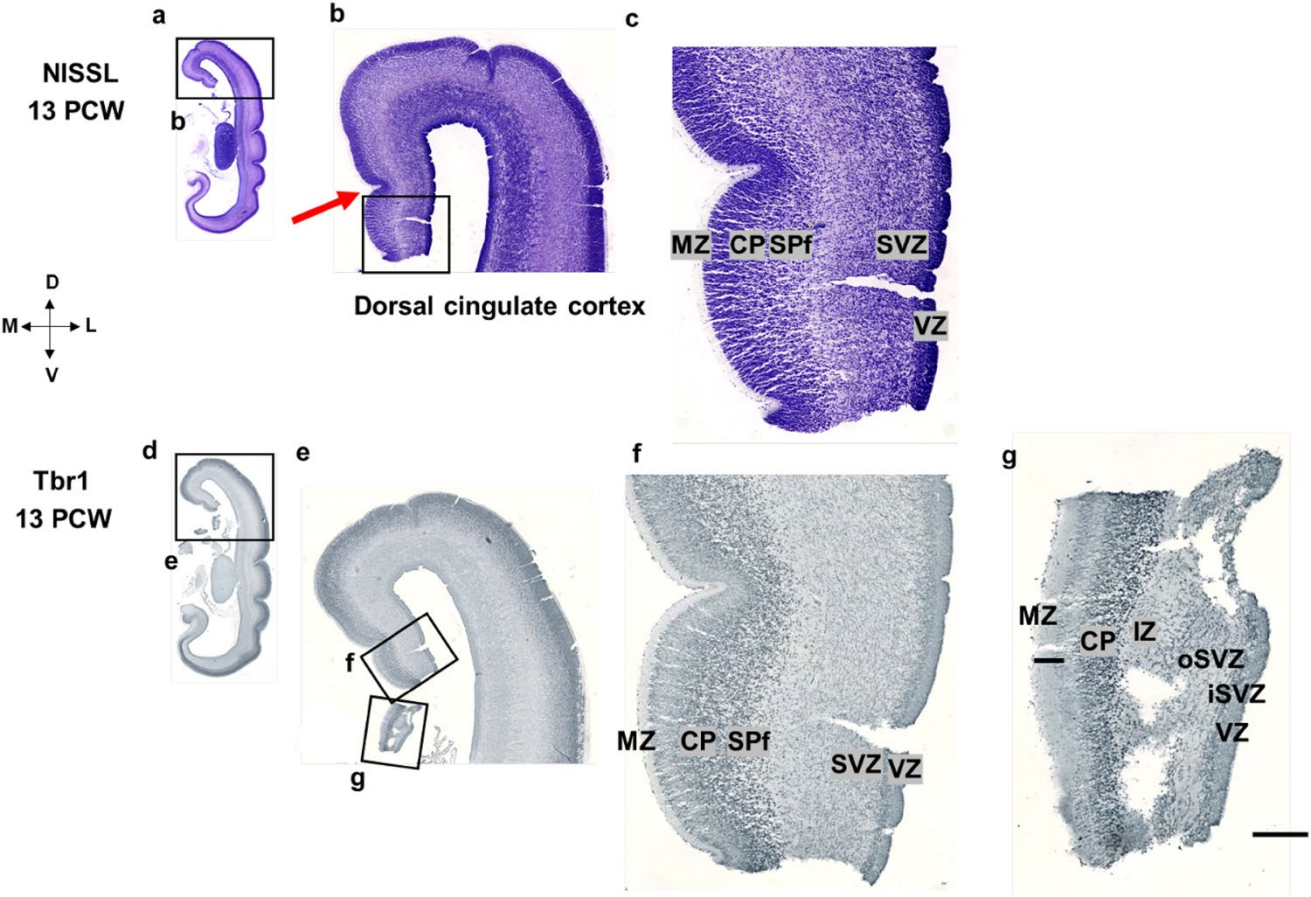
Dorsal and ventral cingulate cortex characteristics (coronal sections, intermediate part—level of the hippocampus) revealed by Nissl staining (**a, b, c**) and Tbr1 immunohistochemistry (**d, e, f, g**). Coronal Nissl-stained section at 13 PCW at the level of the hippocampus (**a**). Magnification from the rectangle shows dorsal cingulate cortex (dorsal pattern) and prospective cingulate sulcus (red arrow) (**b**). Laminar architecture of neocortical dorsal cingulate cortex (c). Coronal section at 13 PCW at the level of the hippocampus shown with Tbr1 marker (**d**). Magnification from the rectangle (**e**) shows the dorsal (**f**) and ventral (**g**) portion of the cingulate cortex. Laminar architecture of the neocortical dorsal cingulate cortex (**f**). Laminar architecture of the mesocortical ventral cingulate cortex: wide MZ, thinner CP and no SP expansion (**g**). Please note that the ventral cingulate portion (**g**) was detached during tissue processing. *MZ* marginal zone, *CP* cortical plate, *SPf* subplate in formation, *SVZ* subventricular zone, *iSVZ* inner SVZ, *oSVZ* outer SVZ, *VZ* ventricular zone. Scalebar: 100 μm

The isocortical (neocortical) SP zone is formed by the secondary expansion of CP cells between 13 and 15 PCW (Duque et al. 2016). Here, regional differences in the SP formation process are observed with Tbr1 marker between the dorsal isocortex and medial interhemispheric cortex composed of the dorsal isocortical and ventral mesocortical portion of the cingulate cortex (Fig. 7). Using Tbr1 immunohistochemistry, we observed the absence of CP delamination (secondary expansion of SP) in the archicortex (Fig. 7). Thus, our main findings reveal how to anatomically differentiate and delineate the dorsal isocortical part of the cingulate gyrus, ventral allocortical-mesocortical part of the cingulate gyrus, and the archicortex (future indusium griseum). The neocortex is characterized by the complete “second” cortical plate (Poliakov 1949) formation and consequently, complete SP formation from the deeper portions of the CP. We correlated these neuroanatomical findings with DPN marker Tbr1 (Figs. 7, 13). Remarkably, the mesocortical part of the cingulate cortex is characterized by the incomplete “second” plate formation; on the contrary, the archicortex has no “second” plate nor the SP formation (Figs. 7, 8). At 13 PCW, CC is formed in the anterior brain portions and it is seen as fibers between iSVZ and oSVZ. The tissue bridge between the two cerebral hemispheres which may correspond to the precallosal “sling” is visible only in some planes of the earliest specimen examined in our study (Shu and Richards 2001).

Notably, proliferative SVZ is thick in the neocortex, narrow in the mesocortical part of the cingulate gyrus, and SVZ is not observed in the archicortex. We suggest that the SVZ thickness is associated with the SP formation and expansion process contributing to differences between isocortical and allocortical development. The SVZ narrowing towards the limbus corresponds to the gradient of diminishment and incomplete SP expansion.

**Fig. 9:**
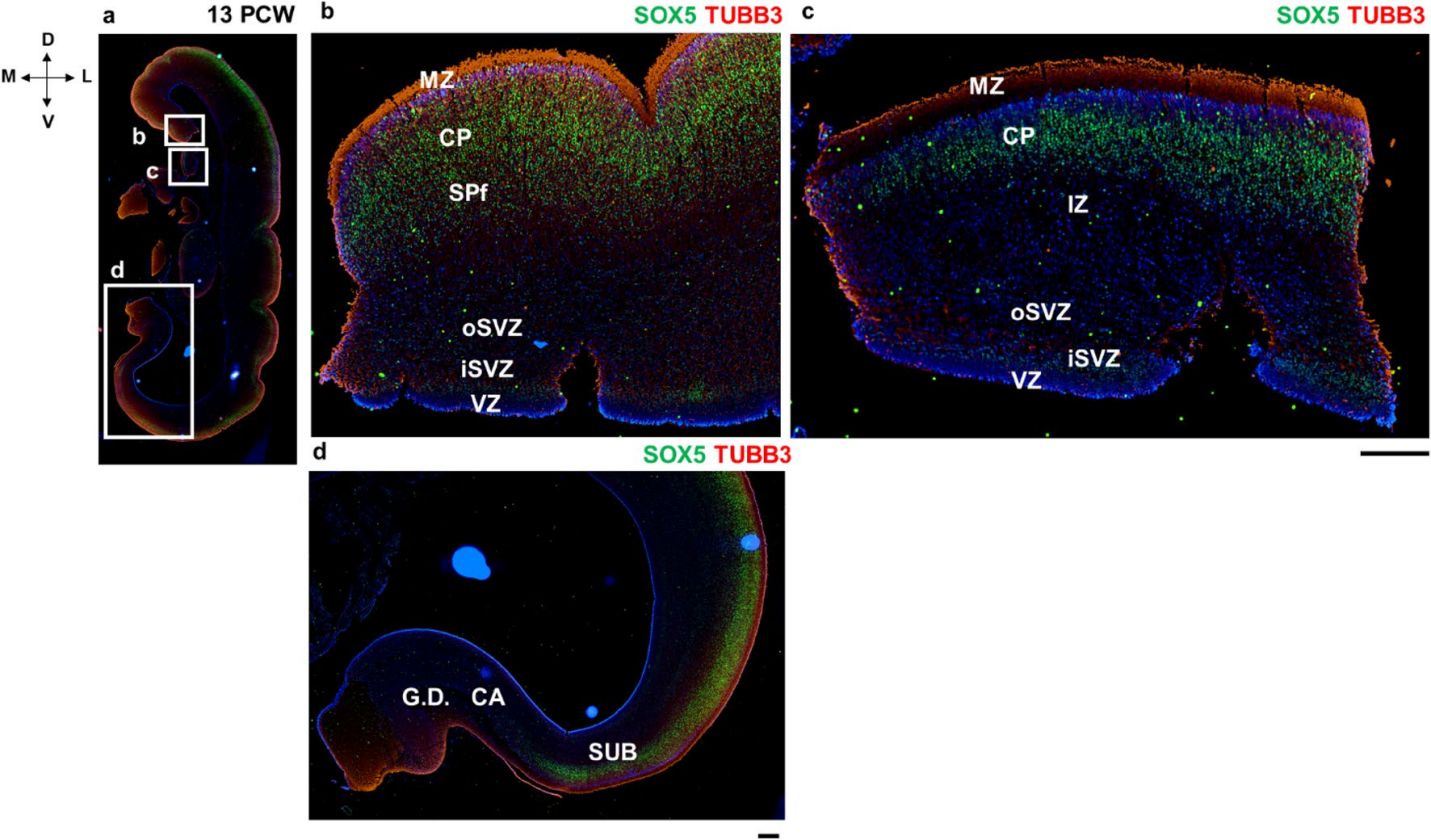
Dorsal and ventral laminar pattern of the medial interhemispheric cortex at 13 PCW (coronal sections, intermediate part—level of the hippocampus) revealed by projection neuron marker SOX5. Dorsal pattern (**b, c**) demonstrates thinning of the CP in the ventral cingulate cortex (SOX5 positive cells). Note that SOX5 marks the border of the mesocortex and archicortex. **b**: dorsal cingulate cortex, **c**: ventral cingulate cortex, **d**: hippocampal formation. *MZ* marginal zone, *CP* cortical plate, *SPf* subplate in formation, *SVZ* subventricular zone, *iSVZ* inner SVZ, *oSVZ* outer SVZ, *VZ* ventricular zone, *G.D.* gyrus dentatus, *CA* cornu ammonis, *SUB* subiculum. Scalebar: 100 μm

Notably, the cingulate cortex and hippocampal formation have a wide and thick MZ. The enlarged MZ of the archicortical hippocampus shares the characteristics of the neocortical SP, such as intensive synaptogenesis, the enlarged thickness of the zone, and an abundant ECM (Vasung et al. 2010). One of our main findings in the hippocampal formation is that Cornu ammonis (CA) sectors of the hippocampus do not show Tbr1 nor SOX5 expression (Fig. 9d). A sharp border between the isocortex and allocortex where CP expansion is absent was observed in the ventral limbic pallium at 13 PCW (not shown).

**Fig. 10:**
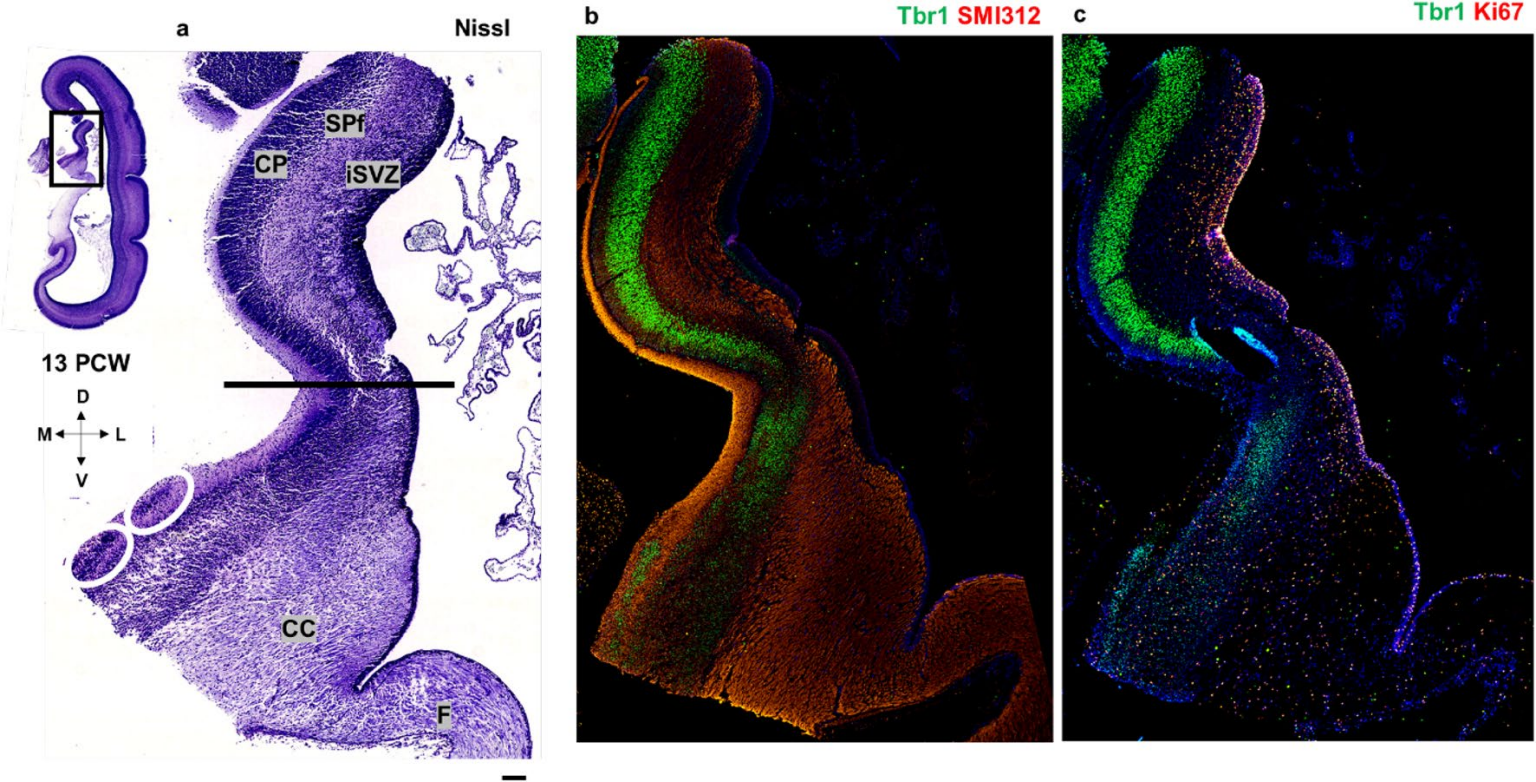
Coronal section through the posterior level of the dorsal allocortex. Hippocampal formation is visible ventrally. At this level, transition towards parahippocampal allocortical pattern is seen—presence of cell islands, dissecant layer and deep principal lamina. Tbr1 is present homogeneously in the neocortical and mesocortical portion of the cingulate cortex, but not in the parahippocampal pattern (Tbr1 positive cell islands). *MZ* marginal zone, *CP* cortical plate, *SPf* subplate in formation, *IZ* intermediate zone, *iSVZ* inner subventricular zone, *CC* corpus callosum, *F* fornix. Scalebar: 100 μm

Using DPN markers, such as markers of the future SP neurons and cortical layer VI and V neurons (Bedogni et al. 2010), we further confirmed the differences in the SP expansion period between the isocortical, mesocortical-allocortical portion of the cingulate cortex, and the archicortex (Fig. 9) in a way that the incomplete SP expansion is observed in the ventral cingulate cortex at 13 PCW. As an additional marker to delineate the border between the transitional mesocortical part of the cingulate gyrus and the archicortex we used a DPN marker SOX5 (Fig. 9). An interesting finding was the transitional future retrosplenial cortex (RSC) where cell islands, dissecant layer and deep principal lamina were observed (Fig. 10).

**Fig. 11:**
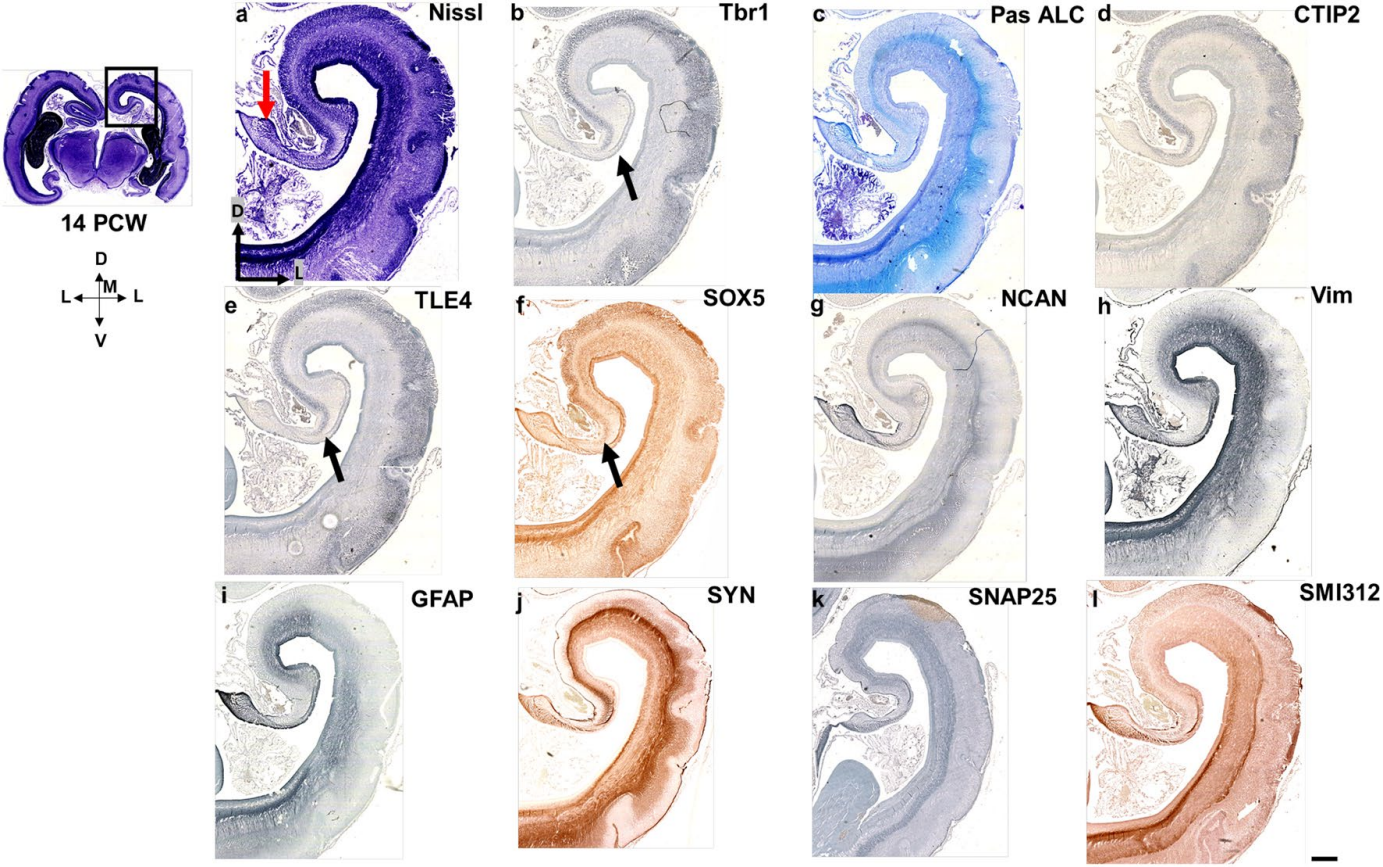
Medial interhemispheric cortex of the cingulate gyrus at 14 PCW (coronal sections, intermediate part) revealed by diverse molecular markers (**a**-l): projection neuron markers Tbr1 (**b**), CTIP2 (**d**), TLE4 (**e**) and SOX5 (**f**), synaptic markers SYN (**j**) and SNAP25 (**k**), ECM marker NCAN (**g**), Vim (**h**) and GFAP (**i**) as intermediate filament proteins and markers of radial glia, and fibrillar marker SMI312 (**l**). Thinning of the CP is observed in the dorso-ventro-medial gradient. The termination of the CP in the archicortex is marked with black arrow (**b, e, f**). Future indusium griseum is marked with red arrow (**a**). Please note that gyrification has not started yet, these are tissue fixation artifacts. Scalebar: 100 μm

**Fig. 12:**
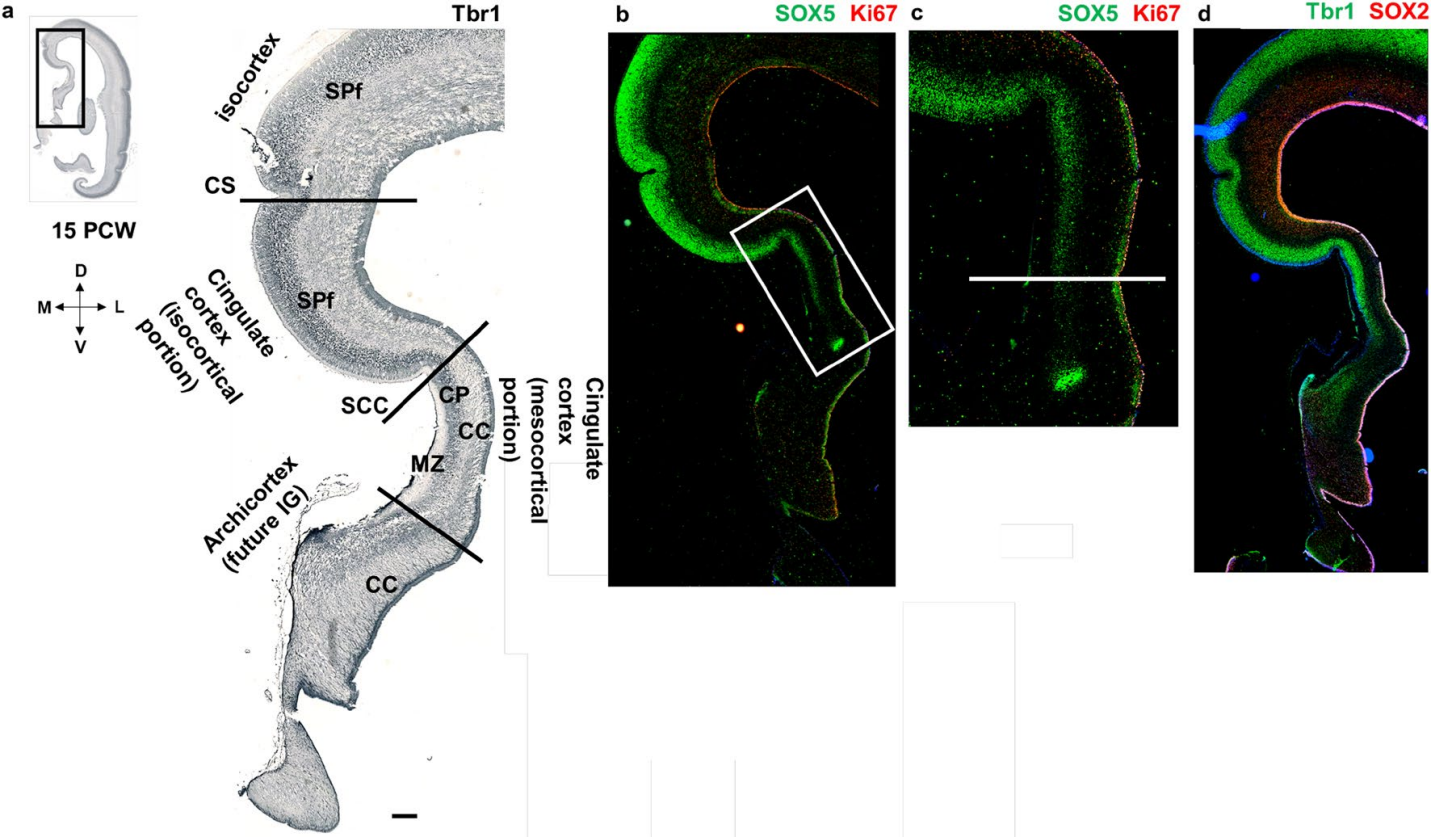
Medial interhemispheric cortex at 15 PCW (coronal sections, posterior level) revealed by projection neuron markers. Topographical relations of the cingulate cortex resembling later fetal topography for the first time during development (appearance of the cingulate sulcus and sulcus of the corpus callosum). Mesocortical portion of the cingulate cortex is characterized by a wide MZ, thinner Tbr1 positive CP and gradually thinner SVZ. Above the corpus callosum, archicortex (future indusium griseum) is observed. Deep projection neuron marker, Tbr1 (**a, d**), demonstrates the differences in SP expansion of the neocortex, mesocortex (SP expansion is incomplete) and archicortex (SP expansion is absent). SOX5 (**b, c**), marker of cortical layer V neurons, serves to delineate neurons of the layer V, as a key element of the cingulate cortex. Ki67 marks proliferative cells. SOX2, stem cell marker, is expressed in the VZ and SVZ (**e**). *MZ* marginal zone, *CP* cortical plate, *SPf* subplate in formation, *SVZ* subventricular zone, *VZ* ventricular zone, *CC* corpus callosum, *CS* cingulate sulcus, *SCC* sulcus of corpus callosum, *IG* indusium griseum. Scalebar: 100 μm

We showed that a transcription factor Tbr1 is a reliable indicator of CP delamination, whether the CP is complete or incomplete, and SP formation-expansion (Figs. 7, 11, 12). Our findings suggest that in addition to other criteria, the grade of the SP expansion is one of the main landmarks for delineating the isocortex from the allocortex during cortical development, especially in the period from 13 to 15 PCW.

**Fig. 13:**
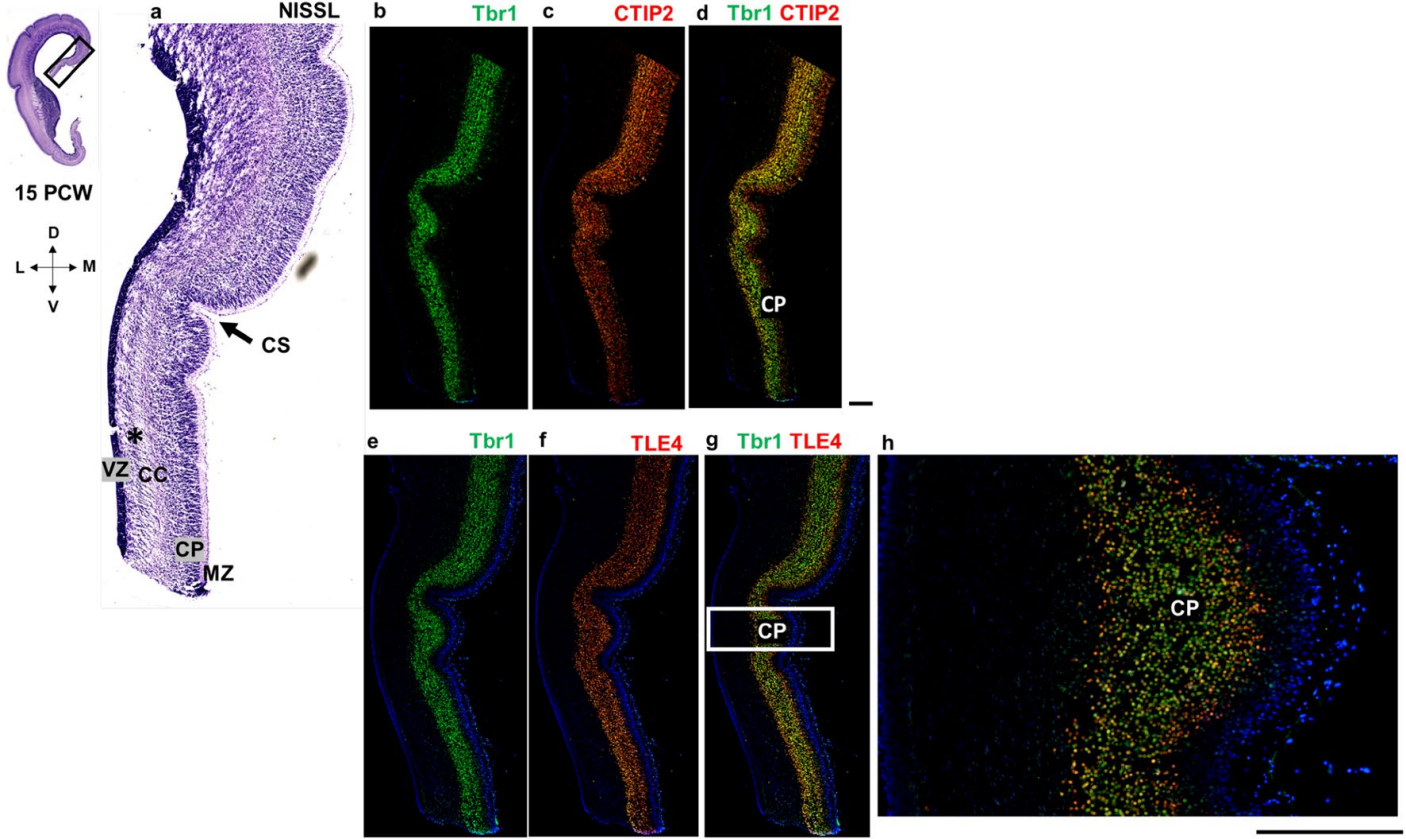
Medial interhemispheric cortex (coronal sections, anterior part) at 15 PCW shown with Nissl staining (**a**) and molecular markers (**b–h**) on adjacent sections. All transient cortical compartments are present. Black arrow shows prospective cingulate sulcus. Projection neuron markers Tbr1 (**b**) and CTIP2 (**c**) show future cortical layer V and VI neurons, as well as SP neurons. Colocalization of Tbr1 and CTIP2 (**d**) is observed in the CP (yellow). Colocalization of Tbr1 and TLE4, projection neuron marker for future cortical layers VI and V (**g**), is observed in the CP (magnification of the CP: (**h**)). Inner subventricular zone is marked with the black asterisk. *MZ* marginal zone, *CP* cortical plate, *iSVZ* inner subventricular zone, *VZ* ventricular zone, *CC* corpus callosum, *CS* cingulate sulcus. Scalebar: 100 μm

At 15 PCW, topographical relations resemble late fetal period topography (> 24 PCW) of the cingulate gyrus for the first time during cortical development (Fig. 12). Cingulate sulcus, neocortex with enlarged SP expansion, sulcus of the CC, and the transitional mesocortex in the depth of the sulcus are observed (Fig. 12). Mesocortex shows enlarged MZ and thinner CP, while SVZ gradually disappears. Above the CC, the archicortex (future indusium griseum) is observed (Fig. 12). It is important to emphasize that the future indusium griseum-archicortical portion of the allocortex covering callosal fibers, can be observed only when CC is already developed (at 10–10,5 PCW in the anterior parts of the brain). Additionally, at 15 PCW, CP and SP are developed and colocalization of DPN markers Tbr1 and CTIP2 is observed in the CP (Fig. 13).

**Fig. 14:**
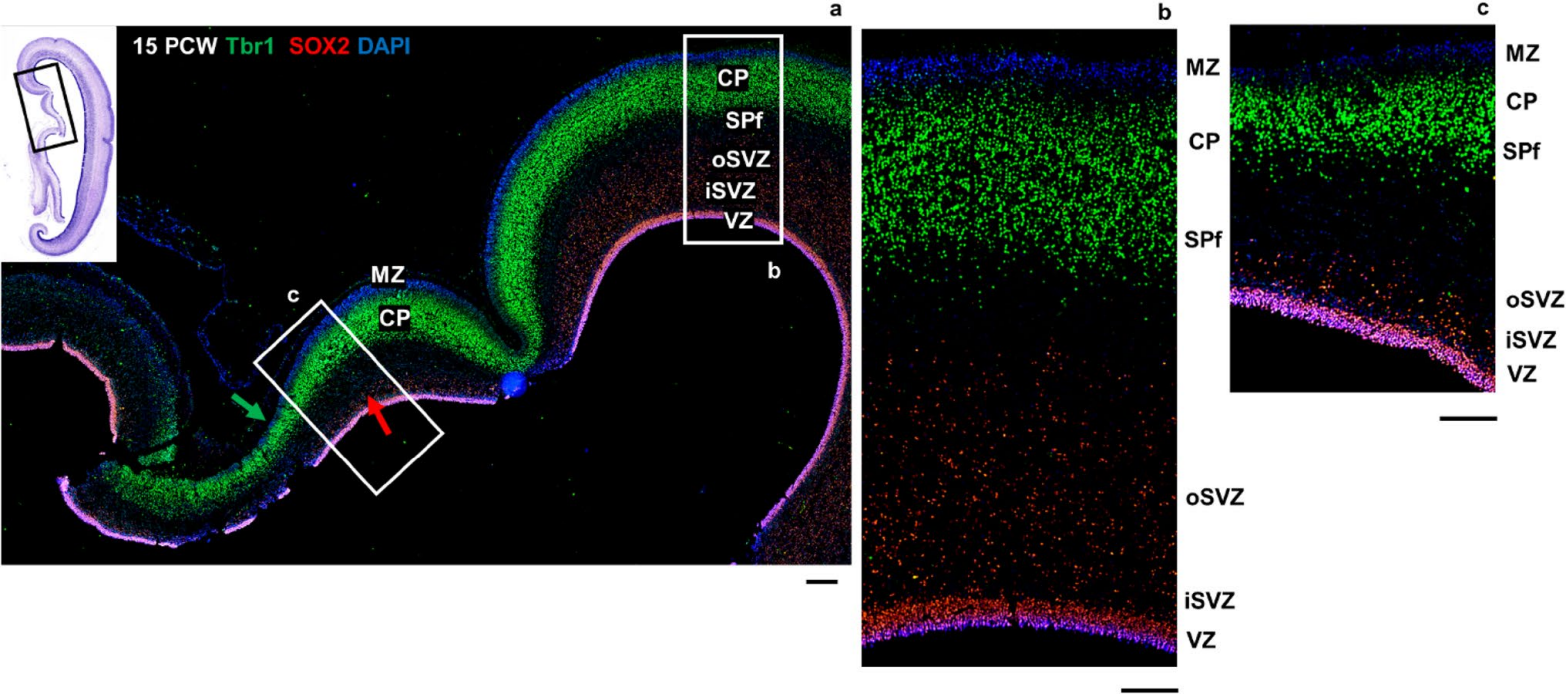
Medial interhemispheric cortex at 15 PCW (coronal sections, posterior part) revealed by a projection neuron marker Tbr1 and a stem cell marker SOX2. Narrowing of the Tbr1 positive CP (green arrow) and SOX2 positive SVZ (red arrow) is observed in the cingulate cortex (**a**). SOX2, stem cell marker, is expressed in the VZ and SVZ, while projection neuron marker Tbr1 is expressed in the CP and SPf. Higher magnification of the SP formation process reveals the isocortical (**b**) and mesocortical pattern (**c**) of SPf. Mesocortical pattern of SPf demonstrates thinning of the CP and SVZ and incomplete SP expansion (**c**). *MZ* marginal zone, *CP* cortical plate, *SPf* subplate in formation, *iSVZ* inner subventricular zone, *oSVZ* outer subventricular zone, *VZ* ventricular zone. Scalebar: 100 μm

In conclusion, we delineated the dorsal from the ventral cingulate cortex (isocortical from mesocortical-allocortical) in a way that the ventral cingulate cortex is characterized by the thinner CP, incomplete formation-expansion of the SP, and typically enlarged MZ (Figs. 7, 9, 14). We showed these cytoarchitectonic differences of the SP expansion (CP delamination) with DPN markers (Tbr1, CTIP2, TLE4, SOX5), while the narrowed SVZ towards the limbus was shown with an intermediate progenitor marker Tbr2 and stem cell marker SOX2. The width of the fibrillar MZ in the mesocortical part of the cingulate cortex is increasing towards the limbus, while DPN containing CP is narrowing together with the disappearance of the PSP zone and gradual diminishment of the SVZ (Fig. 14). According to our results, SP formation-expansion pattern is a crucial event in the isocortical part of the cingulate cortex, while the main characteristic of the mesocortical belt is an incomplete CP delamination and incomplete SP expansion, clearly demonstrated with DPN markers (Fig. 14).

## Discussion

Using immunocytochemical DPN visualization and analysis of their differential laminar dynamics in the fetal cerebral compartments, we have shown that the mesocortical, ventral (allocortical) portion of the future cingulate cortex can be distinguished from the larger, dorsal (isocortical) portion of the future cingulate cortex immediately after the formation of the cortical plate at 8 PCW, before the corpus callosum formation. The DPN markers’ expression pattern, as well as analysis of all proliferative, migratory, and differentiation compartments of the cerebral wall (VZ, SVZ, IZ, SP, CP, MZ) during this early fetal period, serves also as a reliable tool in the delineation of the mesocortex apart from the very limbic (dorsal) archicortex. The early laminar DPN settlement suggests that DPN migrate radially along glial fibers (Arellano et al. 2021) from SVZ to the CP and establish regional differences several weeks before interaction with thalamic afferents and several months before cytoarchitectonic areal differentiation of Brodmann’s type (Filimonoff 1947).

During the SP formation between 12 and 14 PCW, the neocortical cingulate cortex portion displayed complete SP expansion, while the mesocortical portion showed incomplete CP delamination and minor SP expansion. We propose this event as a novel criterion in defining the regional geography of the early developing cingulate cortex present several months before differentiation in the Brodmann type of arealization, as described for mature cingulate cortex by Vogt et al. (1987, 1995). Finally, our study supports the findings that molecular and transcription factors regulating the DPN identity in the cingulate cortex, are instrumental during the first trimester of intrauterine life in humans (Kwan et al. 2012; Willsey et al. 2013). Importantly, developmental disorders involving projection neurons and their circuitry may emerge during this critical period. In the following paragraphs, we further discuss these three aspects of our findings.

### Early establishment of the cingulate cortex-type specific differentiation by deep projection neuron marker laminar organization

Current studies of initial cortical mantle parcellation in the mammalian cerebrum were focused on the early cortical maps formation (protocortex) finding clear differences between medial (hem) and lateral cortical mantle and establishment of anteroposterior cortical gradient and molecular specification (Rubenstein and Rakic 1999; Fukuchi-Shimogori and Grove 2001; O’Leary et al. 2007; Cadwell et al. 2019). These findings were in accordance with Rakic radial unit hypothesis (Rakic 1988, 2000). Rakic documented that the cell production mosaic in proliferative zones and subsequent organization along radial glia (Arellano et al. 2021) towards CP is a basic principle in the formation of the embryonic columns, thus explaining a gradual cortical maps formation by molecular cellular events in the VZ and SVZ (Nowakowski et al. 2017). According to many studies, Brodmann type of areal specification is formed by the additional influence of cortex-type specific thalamic input (thalamocortical afferents) to cortical neurons (O’Leary et al. 2007; Martini et al. 2018; Simi and Studer 2018; Cadwell et al. 2019).

However, classical neuroembryologists (Rose 1926; Filimonoff 1947; Stephan 1975) showed that in the human cortex, there is a long period (approximately three months) between the initial protomap appearance (Clowry et al. 2018) and the Brodmann maps establishment. During this period, the cortex is developed in basic types (allocortex, isocortex) (Rose 1926, 1927a, 1927b; Filimonoff 1947; Stephan 1975) while the ontogenetic formation of classical cortical maps develops rather late during the preterm period, after the appearance of the six-layered Grundtypus (Kostović and Judaš 2002). This classical concept of early cortex-type specific regions (isocortex, allocortex) differentiation into basic histogenetic subdivisions (Economo and Koskinas 1925; Rose 1926, 1927a, 1928; Filimonoff 1947; Stephan 1975)—“Histogenetische Grundgliederung”, (for review see Stephan (1975)), is supported by our results and may explain how cingulate neocortex and mesocortex appear in belt-like shape along the limbus of the medial interhemispheric mantle, surrounding the dorsal archicortex (choroidal fissure).

First, DPN laminar dynamics show differentiation between mesocortex and neocortex in proliferative (SVZ), migratory (IZ), and postmigratory (CP) cortical compartments. SVZ is thinner in the mesocortex (Kostović and Krmpotić 1976; Pogledic et al. 2021), CP shows wedge-shaped narrowing, while synapse-rich MZ is enlarged towards the archicortex (Kostović and Krmpotić 1976; Kostović et al. 2004). There is no CP that early during development (Kahle 1969; Stephan 1975; Kostović-Knežević et al. 1988; Supèr et al. 1998). We emphasize that this basic cortex-type architecture is “designed” predominantly by the DPN layer, given that superficial projection neurons are not born yet at this early time (Rakic 1974, 1988).

Second, we proposed that the deep CP expansion and SP formation between 13 and 14 PCW can be used as a novel, previously not applied criterion for histogenetic cortex-type specific divisions. The deep loose CP expansion (Kostovic and Rakic 1990; Duque et al. 2016) occurs in the cingulate isocortex in a similar mode as in the dorsolateral neocortex, while the allocortical CP expansion is incomplete, especially at the archicortical border. Important evidence that early cortex-type specification is related to the development of connectivity was presented by Kostović and Krmpotić (1976) and Kostovic et al. (Kostović et al. 2004). Namely, in the neocortex, synapses were predominantly distributed in the MZ and poorly delineated CP, together with the presubplate (PSP) containing only a few synapses. On the other hand, in the dorsal isocortical cingulate cortex, the majority of synapses were present in the deep SP. In the early cingulate cortex, Tbr1 reactivity was present in the whole CP thickness. However, SOX5, a marker of prospective cortical layer V neurons, was present in the superficial portion of the CP, indicating a prospective position of future cortical layer V. Deep expanded CP-SP complex showed a predominance of CTIP2 and TLE4 layer-enriched markers, suggesting common laminar dynamics of layer VI and SP.

Throughout the period examined in the present study, the deep Tbr1 immunoreactive nuclei distribution outlined the shape of the thin CP in the medial cortex in the early fetal phase (8–10 PCW). The most superficial Tbr1 and Reelin immunoreactive neurons of Cajal-Retzius type, nicely show gradual enlargement of the MZ towards the limbic archicortex (Meyer et al. 2000; Bayatti et al. 2008; Alzu’bi and Clowry 2019). However, after the great commissure CC is within the commissural plate, (Hochstetter 1919; Rakic and Yakovlev 1968; Stephan 1975) around 10 PCW (CRL around 50 mm), Tbr1 reactive nuclei were present in the SVZ along with a proliferative cells marker (Ki67), and intermediate progenitors marker (Tbr2). To our knowledge, the period of early CC development between 10 and 14 PCW was not previously described with markers specific to the intermediate progenitors` identity. The most interesting finding was the presence of well-developed SVZ during an early period when DPN are being produced for the limbic cingulate cortex in the primate brain (Rakic 1974; Rakic and Nowakowski 1981). Notably, many layer VI DPN and SP neurons project to the primate-characteristic thalamic associative nuclei, such as the mediodorsal (MD) nucleus (Goldman‐Rakic and Porrino 1985).

Accordingly, it seems logical that the SVZ plays a major role in similarly building primate-characteristic circuitry as proposed for a thoroughly examined role of SVZ during the late second trimester (Rubenstein and Rakic 1999; Hansen et al. 2010; Dehay et al. 2015; Nowakowski et al. 2017). SVZ prominence is an additional excellent indicator of the mesocortical cingulate thickness, which diminishes towards the limbus, parallel with the CC development. Thus, our study further supports the general concept of the SVZ importance for primate cortex formation (Rakic and Nowakowski 1981; Haubensak et al. 2004; Hevner 2007; Kelava et al. 2012; Dehay et al. 2015; Popovitchenko and Rasin 2017; Mora-Bermúdez and Huttner 2022). The fact that the primate characteristic SVZ is producing DPN—predominantly of pyramidal type, shows the tremendous importance of pyramidal neurons for the basic cortical architecture establishment in humans (Cajal 1911; Marin-Padilla 1983; Haydar et al. 2000). This seems to be important for the early cingulate cortex connectivity because pyramidal neurons stretch to the MZ where they form a terminal bouquet in contact with tangential afferents originating from different cortical and subcortical sources (Kostović and Krmpotić 1976; Marín-Padilla 1998). On the other hand, basal dendrites reach deep synaptic stratum and SP neurons dendritic arborization (Bourgeois et al. 1994; Rakic et al. 1994; Kostović 2020).

However, the most enigmatic question regarding thinning of the limbic allocortex is rarely discussed in current studies, including the underlying mechanisms leading to this phenomenon. It is obvious that this limbic (allocortical) portion of the cerebral interhemispheric wall develops in an arc-like fashion above and around a choroidal fissure (Hochstetter 1919; Stephan 1975; O’Rahilly and Müller 2008). It is nicely illustrated on images of medial aspects of hemispheres by Economo and Koskinas (1925), Figure 121 in Stephan`s Handbook (Stephan 1975), Hochstetter models (Hochstetter 1919), and Macchi (Macchi 1951). However, we think that during later development, the crucial morphogenetic factor is the massive interhemispheric callosal fibers formation. First, CC fibers form a barrier for migratory neurons that are produced in the VZ and inner SVZ (iSVZ) and practically split the SVZ. Some of the neurons that do not migrate may stay in the subcallosal gray (Kostović et al. 2002). At the medial limbus of the brain hemisphere, especially during the early developmental period, there may be different signaling molecules acting at the key boundaries between different cell compartments. Therefore, anatomical distinctions might relate to the expression pattern of the transcription factor Lhx2 which is known as a forebrain hem system development regulator (Roy et al. 2014). This mechanism may also be involved in the mesocortical thinning observed in our study. The second proposed mechanism is active signaling and glutamate release in the extracellular space of the VZ/SVZ border that may regulate proliferation (LaMantia 1995). The major objection to this proposal is that during the period of DPN production and migration, the CC is poorly developed, and thus the CC morphogenetic mechanisms may be more important during the second part of gestation (Žunić Išasegi et al. 2018), i.e., during the superficial cortical layers neurons production. By all means, the morphogenetic interactions of signaling molecules such as Lhx2 and ingrowth of callosal axons during changes of proliferative SVZ remain a challenging question for future research.

### Subplate formation (expansion) phase is a hallmark of the human cingulate cortex histogenetic subdivisions before onset of arealization

Previous comparative studies of the human and monkey cortex histogenesis have clearly shown that the synapse-rich SP expansion occurring during the early midfetal period is a hallmark of primate neocortical development (Kostovic and Rakic 1990; Duque et al. 2016; Kostović 2020).

In the present study, we have documented that during the SP formation phase in the human cingulate cortex (12–13 PCW), incomplete SP expansion in the transitional mesocortex is an essential cytoarchitectonic and histogenetic criterion for delineating ventral, mesocortical from dorsal, isocortical cingulate cortex. While in the isocortical cingulate cortex SP expansion—deep CP cells “spread down” (Duque et al. 2016) precedes by neocortical mode, the ventral primordial cingulate belt shows incomplete CP “delamination” and SP is extremely narrow at the border with a curved thin cortical sheet of the supracallosal archicortex—future indusium griseum (“dorsal hippocampus”). This new finding via DPN markers confirms histological observation of Kostović and Krmpotić (1976), synaptic distribution analysis in the interhemispheric cortex (Kostović and Krmpotić-Nemanić 1975; Kostović et al. 2004), structural MRI (Bobić-Rasonja et al. 2021), as well as the concept of the thin SP and MZ enlargement as a characteristic of the limbic allocortex (Kostović et al. 1989; Supèr et al. 1998; Šimić et al. 2022). Using the atypical SP formation process in the ventral cingulate cortex, our study demonstrated that before the cingulate sulcus and callosal sulcus formation, mesocortex occupied a large portion of the interhemispheric cortical mantle (His 1904; Hochstetter 1919; Economo and Koskinas 1925), nicely summarized in Figure 121 by Stephan (1975).

The enlarged MZ with massive tangential afferent input and scanty access to subcortical fibers through PSP may explain fewer synapses in the deep cortex of the mesocortical cingulate portion (Kostović and Krmpotić 1976) and other developing allocortical regions (Kostović et al. 1989). In the adult cerebrum, the mesocortical portion remains as a very thin belt-like territory (Stephan 1975; Bobić-Rasonja et al. 2019). With the subsequent cingulate neocortical portion development, the cingulate sulcus appearance, the mesocortical banding, and neuronal production in the late neocortical SVZ, there is a significant increase in isocortical cingulate size and thickness. Parallel development of thalamic and other subcortical afferents, the entrance of collaterals from the thick cingulum bundle, and later cingulate cortex development are dominated by neocortical connectivity mode and neocortical cingulate white matter differentiation (similar to adjacent areas of the interhemispheric neocortex). In parallel with the process of white matter differentiation and SP expansion, there is a gradual differentiation in broad cytoarchitectonic areas corresponding to subgenual, anterior, intermediate, posterior, and retrosplenial portions of the cingulate cortex. This process is less explored and requires future studies implementing novel molecular markers, not only for deep but also for superficial cortical layers, as well as specific markers for unique fields, such as gigantopyramidal field (Braak and Braak 1976) and unique neuronal classes such as von Economo neurons (Allman et al. 2010).

The SP formation timing in our study is in accordance with earlier and faster timing of events in the limbic cortex in comparison with the more dorsal and lateral neocortex. From the study of cortical neuron birthdating (Rakic 1974, 1988), it is obvious that limbic cortical neurons production begins earlier and finishes faster than neurons from dorsal and lateral neocortex. This phenomenon may be related to the early cingulate cortex involvement in visceral, behavioral, and emotional networks that are expressed already in the human newborn, essential for survival and newborn-mother interaction. It also means that the critical vulnerable period for fine developmental limbic cortex lesions occurs earlier than for the neocortex.

### The developing cingulate cortex circuitry abnormalities underlying neurodevelopmental disorders

Our results show that Tbr1 immunoreactive, presumably glutamatergic DPN are the main architectural and circuitry elements in both isocortical and mesocortical belts in the early human fetal cingulate cortex. During this period, characterized by the intense proliferation in the SVZ, migration, and laminar DPN positioning dynamics, one may expect increased early cingulate circuitry vulnerability leading to a developmental disorder such as ASD. In support of this finding, it is evident that the set of high confidence autism spectrum disorder (hcASD) genes converge (Willsey et al. 2013) on glutamatergic projection neurons in layers V and VI of the human midfetal PFC (Willsey et al. 2013; Bakken et al. 2016). The most connected hcASD gene within this period is found to be Tbr1 (Willsey et al. 2013). Additionally, it is supported by the data highlighting the Tbr1-regulated network of ASD genes in the developing neocortex (Notwell et al. 2016). Somewhat earlier appearance of Tbr1 immunoreactivity in ACC than reported for the medial frontal cortex by Willsey et al. (2013) may be explained by precocious and faster ACC development. Besides the identification of coexpression networks for deep cortical projection neurons, it is essential to define their involvement in synaptic circuitry. The existence of early cingulate synaptic circuitry may also be a possible substrate of abnormalities caused by genetic factors, as well as external influences including hypoxia, ischemia and other prenatal etiological factors. Unfortunately, previous neuropathological studies rarely paid enough attention to possible fine developmental cingulate cortex lesions (Hammarberg 1893; Triarhou 2020), because cingulate functions were not explored during the time of classical neuroembryological cingulate cortex development studies.

We point out that early fronto-cingulate growth and path forming (Vasung et al. 2010) are possible factors in the critical vulnerability period delineation. It is rather surprising that the most common developmental abnormality, CC agenesis, is analyzed for fiber bundles abnormalities (Barkovich and Norman 1988; Koester and O`Leary 1994; Jovanov-Milošević et al. 2009; Edwards et al. 2014), but very little attention was paid to cingulate architecture alternations (Utsunomiya et al. 2006). Possible cingulate architecture abnormalities related to abnormal callosal development are based on the evidence that an expanded deep part of the developing cingulate cortex-SP also sends axons into the CC (Koester and O`Leary 1994; deAzevedo et al. 1997; Jovanov-Milošević et al. 2009).

It remains to be explored whether, when and how early developmental projection neuron lesions in ACC, MCC and PCC may induce changes in the thalamus. Interstitial white matter neurons, subplate neurons (SPN) derivatives, also project to the thalamus (Goldman‐Rakic and Porrino 1985). For understanding the human developmental disorders mechanisms, the PCC and RSC connection, and the connection with the precuneus, related to human characteristic functions such as self-awareness, are all of the special interest (Cavanna and Trimble 2006; Utevsky et al. 2014). In addition, cingulate circuitry abnormalities may cause nociception abnormalities (Vogt 2005). In the context of normal and abnormal functional cingulate cortex development, it is important to note that cingulate hubs are the backbone of the human structural connectome and resting-state activity appears already in early preterm infants (Sporns et al. 2005; Gao et al. 2009; Rubinov and Sporns 2010; Sporns 2011; van den Heuvel and Sporns 2011; Anderson and Thomason 2013; Thomason et al. 2015; Matthews and Fair 2015; van den Heuvel et al. 2018). This developmental period is beyond the phases we have analyzed in the present study. However, alternations of early basic projection circuitry may influence the later circuitry development in supragranular layers, as well as the interneuronal maturation (Greig et al. 2013). Our study gives normative temporal and spatial parameters for normative cellular and laminar data instrumental to tackle the challenging problem of the pathogenesis of behavioral and cognitive neurodevelopmental disorders.

## Data Availability

The datasets generated and analyzed during the current study are not publicly available due to ethical reasons but are available from the corresponding author on reasonable request.

## Abbreviations

ACC: Anterior cingulate cortex
ASD: Autism spectrum disorder
BA: Brodmann area
CA: Cornu ammonis
CC: Corpus callosum
CP: Cortical plate
CRL: Crown-rump length
DPN: Deep projection neurons
DTI: Diffusion tensor imaging
ECM: Extracellular matrix
G.D.: Gyrus dentatus
GW: Gestational weeks
hcASD: High confidence ASD genes
IG: Indusium griseum
iSVZ: Inner subventricular zone
IZ: Intermediate zone
MCC: Midcingulate cortex
MRI: Magnetic resonance imaging
MZ: Marginal zone
oSVZ: Outer subventricular zone
PCC: Posterior cingulate cortex
PCW: Postconceptional weeks
PFC: Prefrontal cortex
PPL: Preplate
PSP: Presubplate
RRID: Research resource identifier
RSC: Retrosplenial cortex
SP: Subplate
SPf: SP in formation
SPN: Subplate neurons
SVZ: Subventricular zone
Tbr1: T-box brain 1
VZ: Ventricular zone
